# Regulation of DNA damage repair and lipid uptake by CX_3_CR1 in epithelial ovarian carcinoma

**DOI:** 10.1038/s41389-018-0046-6

**Published:** 2018-05-01

**Authors:** Jia Xie, Hilal Gurler Main, Joelle D. Sacks, Goda G. Muralidhar, Maria V. Barbolina

**Affiliations:** Department of Biopharmaceutical Sciences, University of Illinois at Chicago, Chicago, IL 60612 USA

## Abstract

Failure of currently used cytotoxic chemotherapy is one of the main reasons behind high mortality from metastatic high grade serous ovarian carcinoma. We found that high expression of a receptor for fractalkine (CX_3_CR1) significantly correlated with shorter survival of patients with serous ovarian carcinoma treated with cytotoxic DNA damage chemotherapies, and reduction of CX_3_CR1 expression resulted in sensitization to several DNA damaging modalities, including x-ray radiation and cisplatin. Here, we show that CX_3_CR1 plays a role in double-strand DNA break response and repair by regulating expression of RAD50 by a MYC-dependent mechanism. We demonstrate that downregulation of CX_3_CR1 alone and in a combination with irradiation affects peritoneal metastasis in an organ-specific manner; we show that CX_3_CR1 regulates lipid uptake which could control omental metastasis. This study identifies CX_3_CR1 as a novel potential target for sensitization of ovarian carcinoma to DNA damage therapies and reduction of peritoneal carcinomatosis.

## Introduction

Cancer metastasis remains a largely unsolved health problem worldwide^1^. Among all cancers affecting women ovarian carcinoma is the deadliest gynecologic malignancy^1^. Among all of its subtypes, high grade serous ovarian carcinoma (HGSOC) is the most predominant and lethal.

There are no effective therapeutic options to treat recurrent chemotherapy-resistant metastatic HGSOC. Patients with metastatic HGSOC undergo debulking surgery and first-line cytotoxic chemotherapy with a microtubule stabilizing taxane and a DNA damage-inducing platinum agent. However, intrinsic and acquired resistance to DNA damaging agents remains a barrier for successful application of these powerful drugs^2^. Novel approaches are urgently needed to restore and maintain efficacy of DNA damage therapy to treat this deadly malignancy.

Chemokine receptors are seven transmembrane G protein-coupled receptors that are activated by their ligands (chemokines) resulting in stimulation of the downstream signaling pathways and changes in cell behavior and function. Chemokine receptors serve as key regulators of many disease states, thus, providing a strong rationale for their development as drug targets for various illnesses^3^. Chemokine receptors regulate the metastasis, and, therefore they have been used as anti-cancer drug targets in both pre-clinical and clinical studies^4–6^.

A receptor for fractalkine, CX_3_CR1 is activated by only chemokine ligand fractalkine (CX_3_CL1), which could be present in both secreted and membrane-tethered forms^7,8^. Our previous studies demonstrated that interaction of CX_3_CR1 expressed in ovarian carcinoma cells with membrane-tethered CX_3_CL1 expressed in peritoneal mesothelial cells mediates peritoneal adhesion of disseminating cells^9^. We and others have shown that secreted CX_3_CL1 facilitates ovarian cancer cell proliferation and migration in vitro^9,10^. We have demonstrated that ovarian cancer cell proliferation at metastatic sites is dependent on CX_3_CL1 expressed by parenchyma of intraperitoneal organs and tissues^11^.

Here, we demonstrate that CX_3_CR1 can regulate double-strand DNA break repair in HGSOC cells subjected to therapeutic agents inducing DNA damage, such as x-ray radiation and platinum-based agents. Our studies also suggest that CX_3_CR1 facilitates fatty acid (FA) uptake. Together, these mechanisms converge in promoting omental metastasis in a xenograft mouse model of the disease.

## Results

### Transient downregulation of CX_3_CR1 sensitizes HGSOC cell lines to DNA damage inducing therapies

#### Expression of CX_3_CR1 predicts survival in patients treated with platinum therapy, gemcitabine, and topotecan

**Fig. 1:**
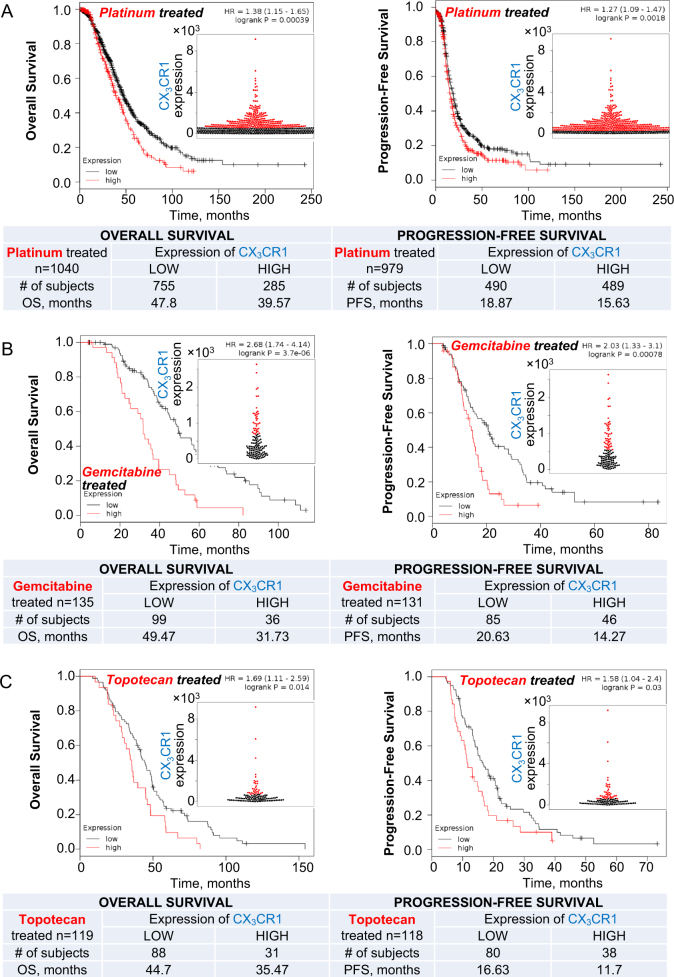
Expression of CX_3_CR1 predicts survival of serous ovarian carcinoma patients treated with DNA damaging agents. High CX_3_CR1 expression predicts shorter overall (OS) and progression-free (PFS) survivals of serous ovarian carcinoma patients treated with platinum therapies (**a**), gemcitabine (**b**), and topotecan (**c**). OS and PFS of serous ovarian carcinoma patients were analyzed using KM Plotter database. Red lines—high CX_3_CR1, black lines—low CX_3_CR1. Numbers of specimens with high (red) and low (black) CX_3_CR1 for each analyzed group and their corresponding average survival are shown in the tables. The best performing threshold was used to determine CX_3_CR1-low and CX_3_CR1-high groups of specimens using KM Plotter software. Expression of CX_3_CR1 in examined specimens is plotted as beeswarm plots shown as inserts; red—high CX_3_CR1, black—low CX_3_CR1. Survival was analyzed with Mantel−Cox log-rank test using KM Plotter software; hazard ratios (HR) and *p*-values are indicated on the graphs

We used Kaplan−Meier Plotter database for serous ovarian cancer^12,13^ to analyze survival of patients treated with the standard of care and second-line chemotherapies, including platinum, gemcitabine, and topotecan, as a function of CX_3_CR1 expression. We have found that both overall (OS) and progression-free (PFS) survivals were significantly shorter when CX_3_CR1 expression was high (Fig. 1). These data suggest that CX_3_CR1 could be considered a biomarker of response to these therapies. Platinum drugs, gemcitabine, and topotecan kill proliferating cells by affecting DNA by different mechanisms; however, one common feature is formation of double-strand DNA breaks (DSBs), which, if not repaired, lead to cell death^14–17^. Hence, these data suggest that CX_3_CR1 may be involved in double-strand DNA repair (DDR).

#### Downregulation of CX_3_CR1 synergizes with x-ray radiation in reducing clonogenic ability of HGSOC

**Fig. 2:**
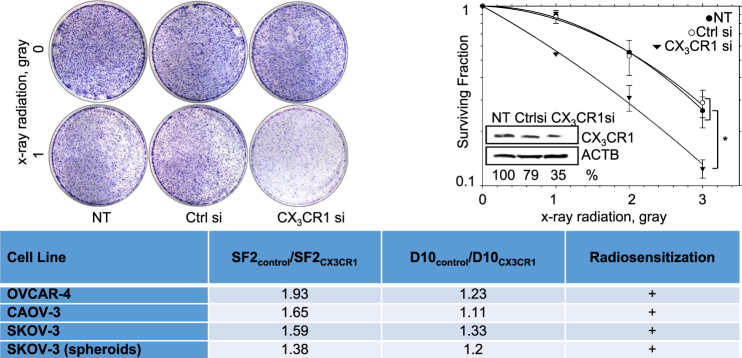
Downregulation of CX_3_CR1 synergizes with x-ray radiation to reduce clone formation of HGSOC cell lines. OVCAR-4 was transiently transfected with either CX_3_CR1-specific (designated “CX_3_CR1si”) or control (designated “Ctrl si”) si RNAs or vehicle (designated “NT”) and subjected to 0, 1, 2, or 3 gray x-ray radiation on the third day following transfection. Images of cells subjected to 0 or 1 gray and subsequently used in clonogenic assay are shown. CX_3_CR1 expression was determined by western blot; ACTB was used as loading control, and normalized expression of CX_3_CR1 was calculated using digital densitometry. Survival curves are the average of five independent experiments. *p < 0.05, two-way ANOVA test. Radiosensitization shown in the table was determined for OVCAR-4, Caov-3, and SKOV-3 (cultured as monolayer and spheroid cultures) if both SF2_control_/SF2_CX3CR1_ and D10_control_/D10_CX3CR1_ were >1.1

To determine the role of CX_3_CR1 in DDR, we used x-ray radiation as a tool to induce DSBs in HGSOC cell lines. Expression of CX_3_CR1 in cell lines OVCAR-4, Caov-3, and SKOV-3 was transiently downregulated using a pool of CX_3_CR1-specific siRNAs. The choice of these cell lines as models of HGSOC was motivated by studies demonstrating that the genomic and proteomic profiles of ovarian carcinoma cell lines OVCAR-4 and Caov-3 bear close resemblance to those of HGSOCs, while proteomic profiling of SKOV-3 suggested that this cell line mimics a more deadly mesenchymal subtype of HGSOC^18,19^. Transient downregulation was given preference to the stable downregulation to avoid reduction of proliferation due to loss of the functional CX_3_CL1/CX_3_CR1 axis, as reported before^9,10^, which would greatly impact the outcomes of the clonogenic assay; instead, our goal was to discern the role of CX_3_CR1 in cellular ability to tolerate DNA damage. Downregulation of CX_3_CR1 in all tested cell lines synergized with x-ray radiation in significantly reducing their clonogenic ability (Fig. 2, Supplementary Figure 1). While the parental cells were able to completely recover from the relatively low doses of radiation (1 gray for OVCAR-4 and Caov-3 and 3 gray for SKOV-3), those with transiently downregulated CX_3_CR1 lost 40−50% of their clonogenic ability. In fact, double dose of radiation (2 gray for OVCAR-4 and Caov-3 and 7 gray for SKOV-3) was required to achieve this level of reduction of clonogenic ability in the parental cells. These data further suggested a potential role for CX_3_CR1 in DNA damage response (DDR).

#### Reduction of CX_3_CR1 leads to formation of persistent γ-H2AX foci and double-strand DNA damage in irradiated cells

**Fig. 3:**
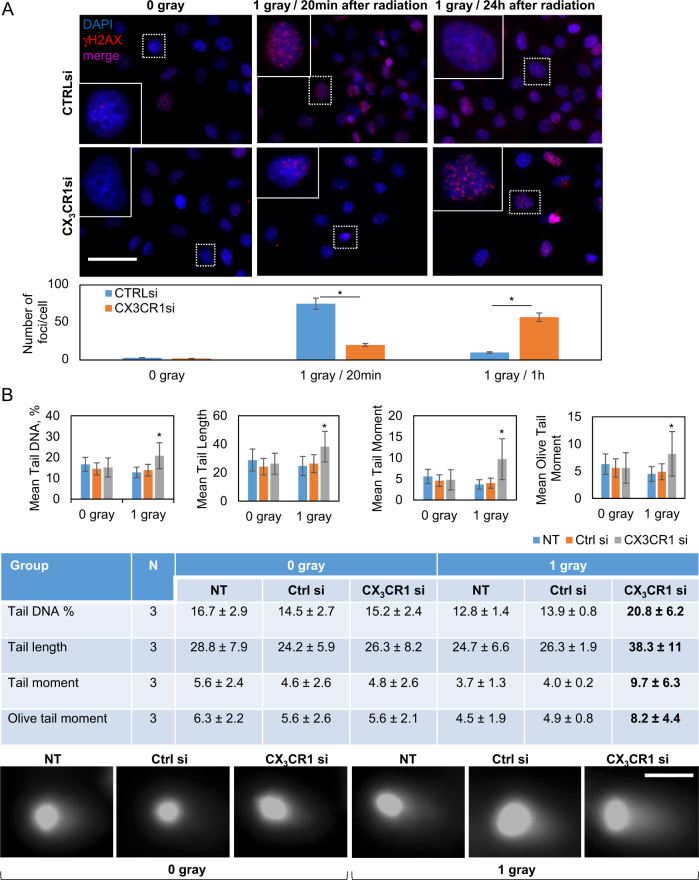
Combination of CX_3_CR1 downregulation and x-ray radiation induces double-strand DNA breaks in HGSOC cell lines. OVCAR-4 was transiently transfected with either CX_3_CR1-specific (designated “CX_3_CR1si”), control (designated “Ctrl si”), or vehicle (designated “NT”) and subjected to 0 or 1 gray x-ray on the third day following transfection. **a** Cells were plated on glass cover slips either 20 min or 24 h after irradiation, as indicated, and stained with γH2AX-specific antibodies. Nuclear DNA was stained with 4′,6-diamino-2-phenylindole (DAPI). Images were superimposed from red and blue channels. Number of γH2AX-positive foci (red fluorescence) in three independent experiments was quantified using Zeiss Axiovert software and plotted. *p < 0.05, Student’s *t* test. Bar, 50 μm. Nuclei outlined with dotted lines were enlarged and shown at corners of the corresponding images. **b** Alkaline comet assay was performed as detailed in Methods. Mean tail DNA (%), mean tail length, mean tail moment, and mean olive tail moment were quantified as an average from three independent experiments, plotted, and tabulated. *p < 0.05, one-way ANOVA and MANOVA tests. Immunofluorescence images (in black&white) show typical nuclei of cells in all tested conditions subjected to comet assay. Bar, 10 μm

To uncover a potential role of CX_3_CR1 in DDR, we determined levels of a phosphorylated form of H2A histone family, member X (γ-H2AX), a surrogate biomarker for DSBs^20^, shortly after (20 min) and 24 h following exposure to x-ray using immunofluorescence staining to define a role of CX_3_CR1 in double-strand DNA damage following irradiation. Although immunoreactivity of nuclear γ-H2AX in cells transfected with control siRNAs was high 20 min post x-ray exposure, significantly fewer γ-H2AX-immunoreactive foci were detected in irradiated cells transfected with CX_3_CR1 siRNAs at the same time point, indicating that detection of the DSBs in irradiated cells with downregulated CX_3_CR1 may have been delayed. In fact, 24 h following radiation exposure, γ-H2AX signal in the nuclei of control cells was very low, indicating that DSBs were successfully repaired. On the contrary, γ-H2AX immunoreactivity was very strong in the nuclei of cells with downregulated CX_3_CR1 (Fig. 3a, Supplementary Figure 2 and 3A), suggesting delayed or impaired DNA damage recognition or repair in these cells. It has been previously suggested that retention of γ-H2AX foci indicates lethal DNA damage^21^. To ascertain that downregulation of CX_3_CR1 coupled with radiation exposure induced DSBs, we conducted a neutral comet assay and found that downregulation of CX_3_CR1 resulted in significant increase in DNA damage in comparison to controls with intact CX_3_CR1 expression (Fig. 3b, Supplementary Figure 3B).

#### Downregulation of CX_3_CR1 results in deactivation of DNA damage repair-related kinases

**Fig. 4:**
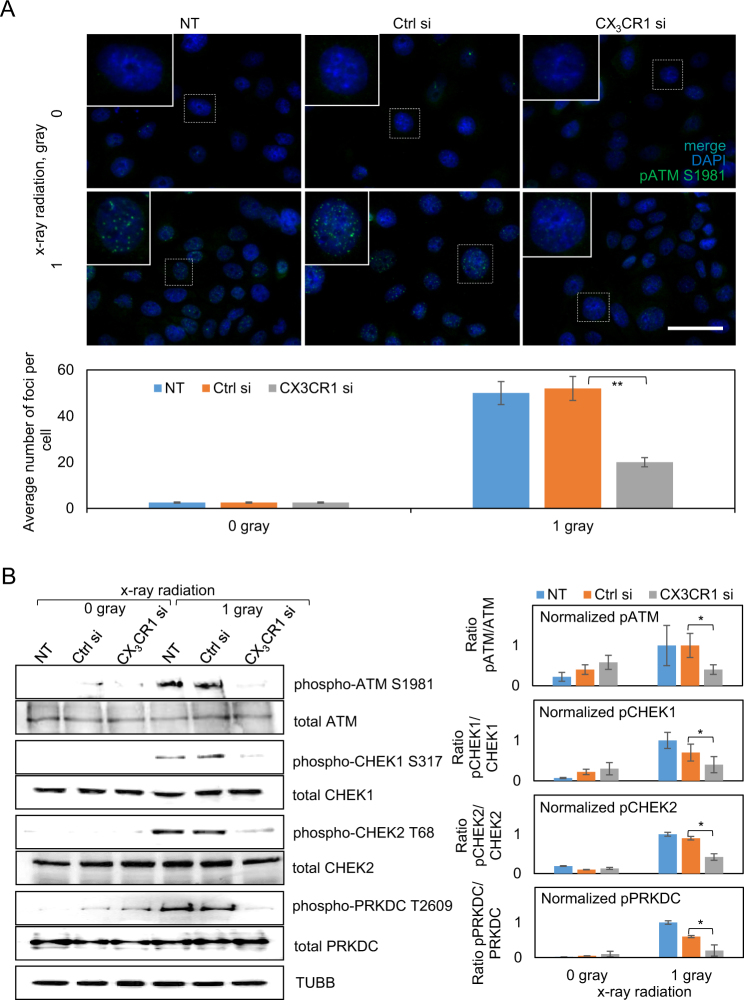
Downregulation of CX_3_CR1 leads to reduced activation of DNA damage repair-related kinases in irradiated HGSOC cells. OVCAR-4 was transiently transfected with either CX_3_CR1-specific (designated “CX_3_CR1si”), control (designated “Ctrl si”), or vehicle (designated “NT”) and subjected to 0 or 1 gray x-ray on the 3^rd^ day following transfection. (**a**) Cells were seeded on glass coverslips. Expression of phosphorylated ATM (at Ser1981) was examined with immunofluorescence staining. Nuclear DNA was stained using DAPI. Images superimposed from blue and green channels are shown. Number of pATM-positive foci (green fluorescence) was quantified for three independent experiments using Zeiss Axiovert software and plotted. *p < 0.05, Student’s *t* test. Bar, 25 μm. Nuclei outlined with dotted lines were enlarged and shown in the upper left corners of the corresponding images. **b** Expression of phospho-ATM (Ser1981), total ATM, phospho-CHEK1 (Ser317), total CHEK1, phospho-CHEK2 (Thr68), total CHEK2, phospho-PRKDC (Thr2609), and total PRKDC was examined with western blot, and three independent experiments were quantified using digital densitometry, averaged, and plotted; TUBB was used as a loading control. Normalized expression of phospho-ATM, phospho-CHEK1, phospho-CHEK2, and phospho-PRKDC was quantified with digital densitometry and plotted. *p < 0.05, Student’s *t* test

It has been shown that downregulation of another chemokine receptor, C-X-C motif chemokine receptor 2, resulted in impairment of ataxia telangiectasia mutated (ATM) kinase activation and decreased intensity of DNA damage response foci in irradiated lung fibroblasts^22^. Hence, we hypothesized that downregulation of CX_3_CR1 in epithelial ovarian carcinoma cells may lead to the same response in irradiated cells. In fact, we found that the number of phospho-ATM foci in irradiated cells with downregulated CX_3_CR1 was significantly lower than in irradiated controls with intact CX_3_CR1 expression (Fig. 4a, Supplementary Figure 4A). Furthermore, addition of an inhibitor of ATM, KU55933, to irradiated cells with downregulated CX_3_CR1 nearly obliterated ATM activation and further radiosensitized cells (Supplementary Figure 5).

As repair of the DSBs caused by ionizing radiation is conducted via both non-homologous end joining (NHEJ) and homologous recombination (HR)^23–25^, we tested activation of DNA damage response kinases, including ATM kinase, checkpoint kinases 1 and 2 (CHEK1 and CHEK2), and DNA-dependent protein kinase, catalytic subunit (DNA-PKcs or PRKDC), using western blot. All of the examined kinases were activated in response to x-ray radiation in the control cells (NT and Ctrl si); however, downregulation of CX_3_CR1 resulted in significantly reduced activation of ATM, CHEK1, CHEK2, and PRKDC in irradiated cells (Fig. 4b, Supplementary Figure 5B,C), suggesting impairment of both NHEJ and HR. These data indicated that irradiation of cells with reduced CX_3_CR1 expression did not activate DNA damage sensor and mediator kinases at the same level as it did in the control cells, suggesting a potential impairment of DNA damage repair pathways along both HR and NHEJ mechanisms.

#### CX_3_CR1 regulates expression of RAD50

**Fig. 5:**
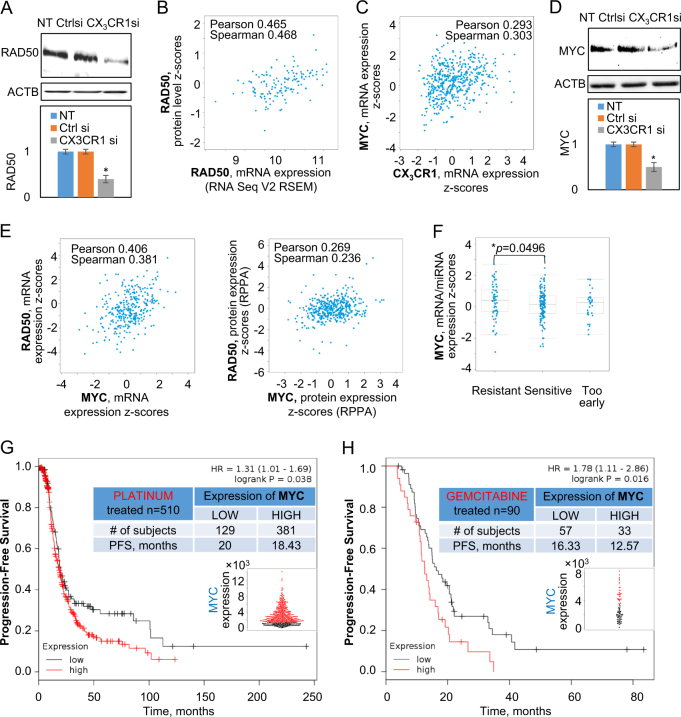
CX_3_CR1 regulates expression of RAD50 and MYC in HGSOC cells. Expression of RAD50 (**a**) and MYC (**d**) in OVCAR-4 transiently transfected with either CX_3_CR1-specific (CX_3_CR1si) or control (CTRLsi) siRNAs or transfected with vehicle (NT) was determined using western blot. ACTB were used as a loading control. Images were quantified with digital densitometry using BIO-RAD Chemidoc software, the results of three independent experiments were averaged and plotted. *p < 0.05, Student’s *t* test. Correlation between expression of RAD50 mRNA and protein (**b**) as well as CX_3_CR1 mRNA and MYC mRNA (**c**) were examined using gene expression data from cBioportal database for serous ovarian cystadenocarcinoma (TCGA, Provisional, *n* = 603); correlation coefficients are indicated on the charts. **e** Correlation between expression of MYC mRNA and protein as well as RAD50 mRNA and protein in specimens of serous ovarian adenocarcinoma were analyzed using cBioportal database and TCGA, Provisional, dataset (*n* = 603); correlation coefficients are indicated on the graphs. **f** Correlation between MYC mRNA expression and platinum response status (resistant, sensitive, too early) in serous cystadenocarcinoma specimens was examined using cBioportal database and TCGA, Nature 2011, dataset (*n* = 557); the data were statistically analyzed using the Mann–Whitney *U* test. OS and PFS of serous ovarian carcinoma patients treated with platinum therapies (**g**) and gemcitabine (**h**) as a function of MYC expression were analyzed using KM Plotter database. Red lines—high MYC, black lines—low MYC. Numbers of specimens with high (red) and low (black) MYC for each analyzed group and their corresponding average survival are shown in the tables. The best performing threshold was used to determine MYC-low and MYC-high groups of specimens using KM Plotter software. Expression of MYC in examined specimens is plotted as beeswarm plots shown as inserts; red—high MYC, black—low MYC. Survival was analyzed with Mantel−Cox log-rank test using KM Plotter software; hazard ratios (HR) *p* values are indicated on the graphs

A protein complex consisting of MRE11 homolog A, double-strand break repair nuclease (MRE11A), RAD50 double-strand break repair protein (RAD50), and nibrin (NBN), or the MRN complex, is one of the complexes responsible for sensing the double-strand DNA damage, activation of ATM, and, ultimately, DNA repair by HR and NHEJ^26,27^. Our data demonstrate that expression of RAD50 was significantly reduced in cells with downregulated CX_3_CR1 expression (Fig. 5a), suggesting that reduction of MRN could be the underlying reason for impaired DDR and reduced clonogenic ability in CX_3_CR1-deficient cells.

RAD50 mRNA expression strongly correlated with the expression of its protein (Fig. 5b), suggesting that it is, at least partially, regulated at the transcriptional level. Several molecules, including V-Myc avian myelocytomatosis viral oncogene neuroblastoma derived homolog (MYCN), myocyte enhancer factor 2C (MEF2C), and V-Myc avian myelocytomatosis viral oncogene homolog A (c-myc; MYC), were demonstrated to regulate expression of RAD50 in different cell types^28–30^. We hypothesized that these mechanisms could play a role in CX_3_CR1-dependent downregulation of RAD50 in ovarian cancer cells. Our studies suggest that MYCN, and MEF2C are not involved in CX_3_CR1-dependent downregulation of RAD50 or other proteins of MRN complex (Supplementary Results 1, Supplementary Figures 6 and 7). MYC was demonstrated to associate with RAD50 gene promoter in chromatin immunoprecipitation studies^28^; moreover, a previous study indicated high nuclear MYC expression in specimens of serous ovarian carcinoma^31^. Since our data demonstrated downregulation of RAD50 expression in cells with downregulated CX_3_CR1 expression (Fig. 5a), we examined a putative role of MYC in CX_3_CR1-dependent expression of RAD50. MYC mRNA expression correlated with expression of CX_3_CR1 in specimens of serous ovarian cystadenocarcinoma (Fig. 5c). Robust downregulation of MYC expression was also observed in cells with downregulated CX_3_CR1 expression (Fig. 5d). Expression of RAD50 (both mRNA and protein) strongly correlated with expression of MYC in specimens of serous ovarian cystadenocarcinoma^32,33^ (Fig. 5e), suggesting that MYC is likely to regulate CX_3_CR1-dependent reduction of RAD50 expression, leading to impairment of both MRN formation and DNA damage sensing. In support, high expression of MYC mRNA significantly correlated with platinum resistance (Fig. 5f) and shorter PFS in platinum- and gemcitabine-treated serous ovarian carcinoma patients (Fig. 5g, h). Consistent with this, downregulation of MYC with specific siRNAs alone and in combination with cisplatin treatment resulted in reduction of cell proliferation in vitro and tumor growth in a xenograft mouse model^34^.

#### Downregulation of CX_3_CR1 synergizes with cisplatin

Cisplatin is one of the standard of care platinum-based agents used to treat peritoneal metastases from HGSOC. Our data analysis demonstrated that both OS and PFS for patients with serous ovarian carcinoma expressing high CX_3_CR1 is significantly shorter than that for patients with low CX_3_CR1 (Fig. 1a). Hence, we characterized the role of CX_3_CR1 in chemosensitization to cisplatin. Our data show that transient downregulation of CX_3_CR1 significantly reduced clonogenic ability of cells subjected to cisplatin and induced moderate, but significant, DNA damage in treated cells (Supplementary Figure 8A−C). Similarly, carboplatin synergized with CX3CR1 downregulation in reducing both cytotoxicity and clonogenic ability (Supplementary Figure 8D, E). Using effect-based approach and the Bliss independence model^35^ we determined that combination index (CI) values were <1, indicating that downregulation of CX_3_CR1 synergized with platinum-based drugs in reducing clone formation of treated cells. Analysis of post-progression survival (PPS) of platinum-, gemcitabine-, and topotecan-treated patients with serous ovarian carcinoma expressing high CX_3_CR1 using KM Plotter database^13^ showed it was significantly lower compared to that for patients with low CX_3_CR1 (Supplementary Figure 9). These data indicate that CX_3_CR1 remained to be one of the determinants of survival and chemotherapy response even after the disease recurred, suggesting that impairment of CX_3_CR1 could potentially re-sensitize recurrent platinum-resistant disease cases.

### Transient downregulation of CX_3_CR1 alone and in a combination with x-ray radiation affects distribution of metastatic lesions at secondary intra- and retro-peritoneal sites in mice

**Fig. 6:**
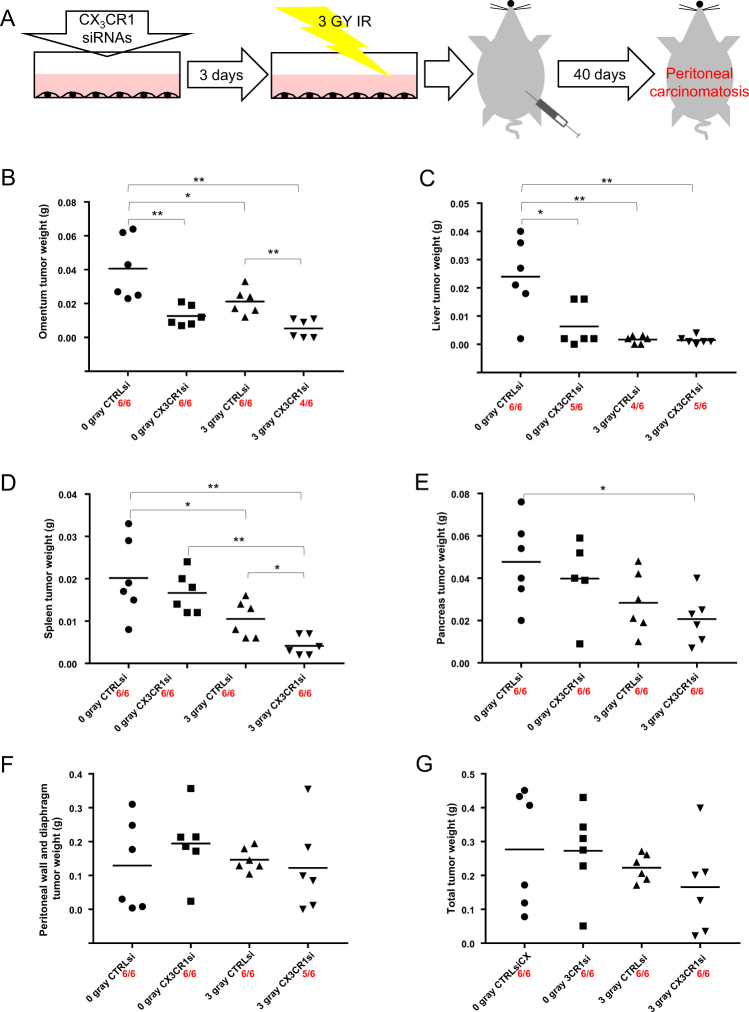
Transient downregulation of CX_3_CR1 coupled with x-ray radiation affects tumor burden at metastatic sites in the peritoneal cavity. **a** A schematic of the experimental setup depicting the sequence of experimental steps: SKOV-3 cells were transiently transfected with CX_3_CR1-specific siRNAs and subjected to 3 gray x-ray on the third day after transfection, cells were collected immediately and injected into the abdomens of athymic nude mice; tumors were allowed to form for 40 days. Tumors formed on the omentum (**b**), liver (**c**), spleen (**d**), pancreas (**e**), peritoneal wall and diaphragm (**f**), as well as the total burden (**g**), as indicated, were collected, weighed, and weights plotted on the graphs. The data were statistically analyzed between all groups with one-way ANOVA; the data between two groups were analyzed using Tukey HSD, Scheffe, Bonferroni and Holm, and Mann−Whitney *U* tests; * *p* < 0.05, **p < 0.001. The number of animals presenting with metastasis out of total number of experimental animals in each group is shown in red numbers below X-axis

Because x-ray radiation applied to cells with siRNA-downregulated CX_3_CR1 induced long-term effect on cell proliferation and clone formation, we tested whether these effects could impair the ability of cells to develop intraperitoneal tumors in vivo. We transiently downregulated CX_3_CR1 using a pool of specific siRNAs in SKOV-3, a model for the deadliest mesenchymal subtype of HGSOC^22^, which reproducibly forms peritoneal tumors consistent with the pattern seen in patients. Experimental groups included irradiated cells transfected with CX_3_CR1 siRNAs (designated “3 gray CX_3_CR1si”), irradiated cells transfected with control siRNAs (designated “3 gray CTRLsi”), and unirradiated cells transfected with either CX_3_CR1-specific or control siRNAs (designated “0 gray CX_3_CR1si” and “0 gray CTRLsi”, respectively) (Fig. 6a). Consistent with our previous observations and expected pattern of metastatic spread of HGSOC in patients, visible tumors had formed at omentum, peritoneal wall, diaphragm, pancreas, liver, and spleen (Fig. 6b–g).

Irradiation and downregulation of CX_3_CR1, either alone or in combination with each other, affected tumor formation at intraperitoneal organs covered by visceral peritoneum as well as at retroperitoneal organs. Both irradiation and CX_3_CR1 downregulation resulted in significantly reduced tumor burden at omentum; moreover, a combined action of irradiation and downregulation of CX_3_CR1 led to significant reduction of tumor burden as well as the number of animals bearing omental metastasis compared to irradiation alone (Fig. 6b). Transient downregulation of CX_3_CR1 alone significantly reduced tumor burden, while irradiation alone almost completely obliterated tumor burden at the liver (Fig. 6c). Tumor burden at the spleen was not affected by downregulation of CX_3_CR1 alone. However, irradiation alone did significantly reduce spleen tumor burden, while a combination of irradiation and downregulation of CX_3_CR1 resulted in significantly greater reduction of tumor burden compared to radiation alone (Fig. 6d). Irradiation and downregulation of CX_3_CR1 significantly reduced tumor burden at pancreas, while individually both factors did not have any effect (Fig. 6e).

Together, these data indicate that transient downregulation of CX_3_CR1 and x-ray radiation alone as well as their combination affect tumor formation in an organ-specific manner.

### Transient downregulation of CX_3_CR1 results in reduction of fatty acid uptake by ovarian carcinoma cells

The omentum is the most commonly colonized site by metastasizing ovarian carcinoma cells^36,37^. Previous studies have suggested that HGSOC cells use FAs from omental adipocytes as energy for growth^38^. GPCRs are known to play a role in FA uptake^39^; however, it is not yet known whether chemokine receptors, including CX_3_CR1, are involved in this process. In our in vivo data, tumor burden at the omentum was significantly reduced by downregulation of CX_3_CR1 and its combination with radiation. We posited that regulation of FA uptake may be one of the underlying reasons for reduced tumor burden in groups with reduced CX_3_CR1.

**Fig. 7:**
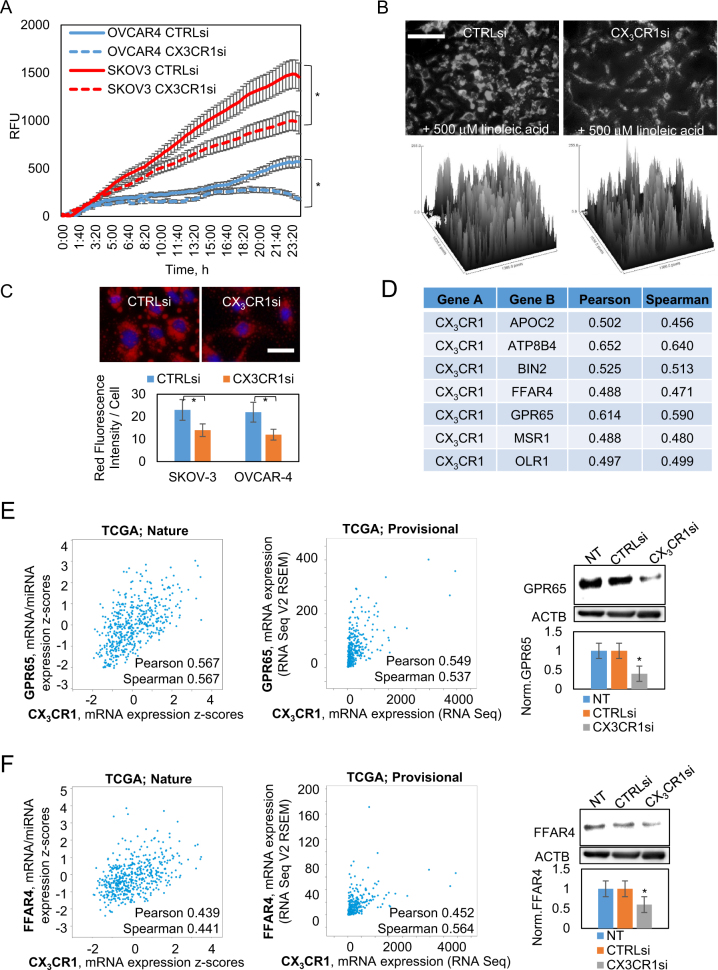
Downregulation of CX_3_CR1 affects fatty acid uptake in serous ovarian carcinoma cell lines. OVCAR-4 and SKOV-3 were transiently transfected with either control (CTRLsi) or CX_3_CR1-specific (CX_3_CR1si) siRNAs and **a** subjected to QBT fatty acid uptake assay. Three independent experiments were averaged and data plotted. *p < 0.05, two-way ANOVA. **b** On the third day after transfection, cells were treated with 500 μM of linoleic acid for 24 h and stained with Oil red O dye and DAPI followed by fluorescence imaging (shown, OVCAR-4). Cells not incubated with linoleic acid were used as controls to determine the baseline fatty acid contents. Bar, 50 μm. All images across the same experiment were imaged using the same exposure time to enable quantitative comparison of immunofluorescence signals. Lipid contents in the cells in the entire field of view were quantified with ImageJ (NIH) software and are shown as surface plots. **c** Images were superimposed from red and blue channels. Three independent experiments were performed with each cell line, ten random fields for each condition and imaged. Fatty acid uptake was quantified by mean fluorescence/cell using ImageJ software and plotted. *p < 0.05, Student’s *t* test. Bar, 20 μm. **d** Genes, as indicated (Gene B column), expression of which strongly correlated with expression of CX_3_CR1 in specimens from patients, as determined using cBioportal database for serous ovarian cystadenocarcinoma (TCGA, Nature 2011 dataset). Expression of GPR65 (**e**) and FFAR4 (**f**) mRNA as a function of CX_3_CR1 mRNA expression in specimens of ovarian cystadenocarcinoma was determined using cBioportal database (left panels: TCGA, Nature 2011 dataset, *n* = 557; center panels: TCGA, Provisional dataset, *n* = 603). Expression of GPR65 and FFAR4 in HGSOC cell lines (shown: SKOV-3) transiently transfected with either control (CTRLsi) or CX_3_CR1-specific (CX_3_CR1si) siRNAs or transfected with vehicle (NT) was determined using western blot (left panels). ACTB was used as a loading control. Data (an average of three experiments) were analyzed with digital densitometry using BIO-RAD Chemidoc software, averaged, and plotted. *p < 0.05, Student’s *t* test

Thus, we tested a role for CX_3_CR1 in FA uptake. We downregulated CX_3_CR1 using a pool of CX_3_CR1-specific siRNAs in OVCAR-4 and SKOV-3. FA uptake was monitored using two approaches: (1) QBT FA uptake assay, (2) incubation with linoleic acid, counterstaining with Oil Red O dye, and fluorescence imaging. Transient downregulation of CX_3_CR1 led to a significant reduction in FA uptake (Fig. 7a−c), suggesting existence of a CX_3_CR1-dependent mechanism of lipid uptake.

We hypothesized that CX_3_CR1 could regulate expression of one of the proteins playing a role in the processes of FA uptake and metabolism. To identify possible partners of CX_3_CR1 in this process, we analyzed co-expression of CX_3_CR1 and genes related to FA uptake and metabolism in patients with serous ovarian cystadenocarcinoma^32,33^. We found that upregulation of CX_3_CR1 mRNA expression significantly co-occurred with upregulation of expression of several genes, including apolipoprotein C2 (APOC2), ATPase phospholipid transporting 8B4 (ATP8B4), bridging integrator 2 (BIN2), free fatty acid receptor 4 (FFAR4), G protein-coupled receptor 65 (GPR65), macrophage scavenger receptor 1 (MSR1), and oxidized low density lipoprotein receptor 1 (OLR1) (Fig. 7d), which regulate FA uptake and metabolism^40–47^. We further investigated regulation of GPR65 and FFAR4 expression by CX_3_CR1. In agreement with data suggesting significant and strong co-expression of CX_3_CR1 with GPR65 and FFAR4 in specimens, western blot confirmed robust downregulation of GPR65 and FFAR4 in ovarian carcinoma cell lines transfected with CX_3_CR1-specific siRNAs (Fig. 7e, f). High expression of both GPR65 and FFAR4 significantly correlated with lower survival in patients with serous ovarian carcinoma (Supplementary Figure 10). Collectively, these data suggest that expression of genes responsible for FA uptake and metabolism, including GPR65 and FFAR4, is, at least in part, dependent on expression of CX_3_CR1. Reduction of CX_3_CR1 expression leads to reduction in GPR65 and FFAR4 expression, which may affect tumor cell’s ability to uptake FAs, partially explaining reduced omental metastasis.

## Discussion

Our in vivo data indicate that transient downregulation of CX_3_CR1 either alone or together with x-ray radiation results in a measurable long-term effect, as it affects organ-specific metastatic colonization of the abdomen. Transient downregulation of CX_3_CR1 significantly reduces formation of lesions at the liver and omentum. At the same time, formation of lesions at the peritoneal wall, pancreas, and spleen is not affected by transient reduction of CX_3_CR1 alone, suggesting that neither attachment to mesothelial monolayer, nor cell growth, nor cell migration and invasion are affected, implying the existence of other yet unknown organ-specific mechanisms supporting metastatic dissemination to the liver and omentum that are abrogated upon transient downregulation of CX_3_CR1. Our studies can at least partially explain the mechanisms involved in CX_3_CR1-dependent reduction of omental metastasis. Our studies indicate that expression of CX_3_CR1 correlates with that of several proteins responsible for FA uptake, and, thus, might contribute to reduced tumor growth. However, it remains to be investigated whether CX_3_CR1 can also participate in direct lipid uptake or regulate other mechanisms leading to reduced omental metastasis.

Interestingly, irradiation alone induced opposite effects on organ-specific colonization. Irradiated cells were not capable of forming tumors at the liver, and colonization of the pancreas, spleen, and omentum was partially reduced, suggesting that radiation treatment can affect formation of recurrent tumors in an organ-specific manner. Together, our data demonstrate that DNA damage induced by irradiation is significant enough to either completely prevent or reduce tumor formation at visceral and retroperitoneal organs. Irradiation is currently used in palliative care of terminally ill ovarian carcinoma patients; hence, patients whose metastases are localized to visceral and retroperitoneal sites may potentially draw benefits from irradiation. Moreover, our studies suggest that downregulation of CX_3_CR1 allows using significantly lower doses of x-ray to achieve the same effects, which may carry a promise of reducing radiation dose and associated toxicities while maintaining the same efficacy in killing tumor cells.

Finally, transient downregulation of CX_3_CR1 together with irradiation brought about the most deleterious effect. Our studies show that dissemination to sites affected by radiation, such as the pancreas, spleen, and omentum, was further negatively affected by CX_3_CR1 downregulation, suggesting that increased DNA damage in cells subjected to both stimuli is the underlying reason for reduced metastatic burden at these sites. Thus, the approach to downregulate CX_3_CR1 together with administration of a DNA damaging agent carries a promise to more effectively kill metastatic cells. However, this approach has to be extensively evaluated using multiple in vivo models and different CX_3_CR1-specific targeting approaches to find the most effective and safest combinations for various patients cohorts. The major side effect of irradiation in treatment of ovarian cancer is irradiation of normal cells, which could be potentially lethal to the patient. Recent studies demonstrated the efficacy of novel methods of radiation delivery that spare normal tissues from radiation damage. Downregulation of CX_3_CR1 expression in this setting could allow reducing the radiation dose and associated toxicities while keeping the efficacy of treatment at the same high level.

Our in vitro studies provide mechanistic explanation for some of the effects seen in the in vivo studies. We show that CX_3_CR1 is instrumental in double-strand DNA damage response and repair by regulating expression of RAD50. Building on these mechanistic studies, one of the most important messages taken from these findings is a potential to use CX_3_CR1 as a target for sensitization to DNA damage chemotherapies frequently used to treat ovarian and other solid cancers. In support, our in vitro studies indicated that cisplatin synergized with downregulation of CX_3_CR1 to reduce clone formation. Importantly, our data indicate that even transient downregulation of CX_3_CR1 in combination with other tested therapies brings about measurable DNA damage resulting in long-term deleterious effects on tumor growth. As CX_3_CR1 is a surface receptor, manipulating MRN expression by targeting CX_3_CR1 could be a promising approach in the future to increase sensitivity to DNA damaging therapies. Hence, it would be important to evaluate combinations of CX_3_CR1-targeting agents with cisplatin and other platinum agents in their efficacy against peritoneal metastasis in future studies.

Collectively, our data suggest that impairment of CX_3_CR1 in ovarian carcinoma could be beneficial from multiple standpoints: (1) it could disrupt FA uptake, further depriving growth of rapidly proliferating metastatic ovarian cancer cells, and, most importantly, (2) it could increase efficacy of DNA damage agents, including x-ray radiation and cisplatin.

## Materials and methods

### Cell lines

Human-derived ovarian carcinoma cell lines OVCAR-4 and SKOV-3 were obtained from National Cancer Institute Tumor Cell Repository (Detrick, MD). Human-derived ovarian carcinoma cell line Caov-3 was obtained from Dr. M.S. Stack (University of Notre Dame, IN). The cell lines were maintained in minimal essential medium or Roswell Park Memorial Institute medium supplemented with 10% fetal bovine serum for no longer than 20 consecutive passages and were routinely assessed by cell morphology and the average doubling time; identity of the cell lines was confirmed using a short tandem repeat analysis that indicated 100% match of cell line-specific DNA loci reported in the ATCC database to the tested samples. The cell lines were free from contamination by *Mycoplasma fermentans*, as was routinely determined using The LookOut Mycoplasma PCR Detection kit (Sigma).

### Mice

Athymic nude FOXN1nu mice were obtained from Harlan Laboratories (Madison, WI). All experimental procedures were performed according to the Institutional Animal Care and Use Committee protocols approved by the Animal Care Committee of University of Illinois at Chicago. Animals were fed ad libitum and maintained in Association for Assessment and Accreditation of Laboratory Animal Care International-approved facilities on a 12 h light−12 h dark cycle.

### Antibodies

Anti-TUBB and anti-ACTB were purchased from Developmental Studies Hybridoma Bank (Iowa City, IA). Anti-rabbit IgG HRP-linked antibody, anti-phospho-CHEK2, anti-phospho-CHEK1, and anti-CHEK1, antibodies were obtained from Cell Signaling Technology (Danvers, MA). Anti- CX_3_CR1, anti-phospho-ATM, anti-phospho-PRKDC, anti-PRKDC, anti-ATM, and anti-phospho-H2AX antibodies were obtained from Abcam (Cambridge, MA). Anti-mouse IgG HRP-linked antibody, anti-phospho-ATM, anti-CHEK2, anti-MYC, and anti-phospho-H2AX were obtained from Santa Cruz Biotechnology (Santa Cruz, CA). Alexa Fluor 488 goat anti-mouse IgG and Alexa Fluor 546 goat anti-rabbit IgG were from ThermoFisher Scientific (Waltham, MA). Anti-GPR65 and anti-FFAR4 antibodies were obtained from One World Lab (San Diego, CA). Anti-RAD50 antibodies were purchased from NeoBiolab (Cambridge, MA).

### Materials and reagents

ECL reagent was purchased from Thermo Scientific (Waltham, MA). Control siRNA, CX_3_CR1-specific siRNAs, and ATM kinase inhibitor were purchased from Santa Cruz Biotechnology (Santa Cruz, CA). Cisplatin was obtained from Sigma-Aldrich (St. Louis, MO). Dharmafect reagent 1 was obtained from GE Dharmacon (Marlborough, MA). QBT^TM^ Fatty Acid Uptake Assay Kit was purchased from Molecular Devices (Sunnyvale, CA).

### Spheroid formation

Plates were coated with 0.5% agarose and allowed to solidify at room temperature for 30 min. 1×10^6^ cells were resuspended in minimal essential media supplemented with 2% fetal bovine serum, transferred on top of the agarose, and incubated at 37 °C at 5% CO_2_ for 48 h.

### Transient transfections

Cells were grown to 70−80% confluency and transfected with siRNA as suggested by the manufacturer using Dharmafect reagent I as a transfection reagent.

### Radiation treatments

Transfected cells as well as controls plated in tissue culture treated 6-well plates were subjected to a single dose x-ray radiation at 0.8 gray/min using a linear accelerator (UIC Radiation Oncology) 3 days following transfection.

### Clonogenic assay

After radiation 1–5% of the cells were replated on 100 mm^2^ diameter tissue culture plates and allowed to grow for up to 2 weeks until visible colonies of more than 50 cells formed. Cells were fixed with 4% para-formaldehyde (PFA) and stained with 0.05% crystal violet solution. Colonies were quantified and used to create survival curves. Surviving fraction of cells was found by detecting OD450 values and plotting using SigmaPlot 12.5 software (SigmaPlot, London, UK). Both quantification methods produced similar results.

### Determination of radiosensitivity

The parameters of the linear quadratic model were used to compare radiosensitivity provided that the data were statistically significant by one-way ANOVA test^48^. Radiation enhancement factor (REF) and dose modifying factor (DMF) were used to compare radiosensitivity between the control and the treatment groups. REF is defined by the surviving fraction of the control group compared to that of the treatment groups at 2 gray (SF2control/SF2treatment). DMF is defined as the dose that is required to kill 90% of the population in the control group compared to that in the treatment group (DMF10control/DMF10treatment). When both REF2 and DMF10 were larger than 1.1, the treatment group was considered to be radiosensitized.

### Immunofluorescence staining

The procedures were performed as we described before^9,11,49,50^. Primary antibodies at 1:50 dilution in 2% goat serum were incubated with cells for 2 h at room temperature. All images across the same experiment were obtained using the same exposure time to enable quantitative comparison of immunofluorescence signals using ImageJ software (NIH).

### Western blot

The procedures were performed as we described before^9,11,49,50^. Anti-CX_3_CR1 and anti-ATM antibodies were used at 1:2000, anti-phospho-ATM was used at 1:5000, anti-FFAR4, anti-RAD50, anti-phospho-CHEK1, anti-phospho-CHEK2, and anti-PRKDC were used at 1:1000, anti-phospho-PRKDC was used at 1:500, anti-CHEK1, anti-CHEK2, anti-TUBB and anti-ACTB were used at 1:200 dilutions.

### Comet assay

Transfected cells and their controls subjected to radiation, or not, were placed on ice immediately after x-ray exposure. Cells were collected, counted, and 2×10^4^ cells were mixed with 1% solution of molten low melting agarose at 1:10 ratio. Cell-agarose complexes were transferred onto agarose-coated glass slides, allowed to solidify, kept at 4 °C for 15 min, and submerged into pre-chilled neutral lysis solution for DSB detection containing 2% sarkosyl, 0.5 M disodium ethylenediaminetetraacetate dihydrate (Na_2_EDTA-2H_2_O), 0.5 mg/ml proteinase K (pH 8.0) for 18 h at 37 °C. Agarose slides were submerged into running buffer containing 90 mM Tris, pH 8.5, 90 mM boric acid, 2 mM Na_2_EDTA-2H_2_O for 30 min with two additional washes followed by DNA electrophoresis at 1 V per cm, wash in deionized H_2_O and ice-cold 70% ethanol, air dried and stained with 1:10,000 dilution of Vista Green DNA Dye (Cell Biolabs, San Diego, CA) for 15 min. Slides were imaged using Zeiss Axiovert fluorescence microscope. At least ten random fields were imaged per each slide. Fifty comets per slide were analyzed using CaspLab.com software.

### Tumor formation

For generation of intraperitoneal tumors 3×10^6^ cells/mouse were injected intraperitoneally (i.p.) into 6−8 week-old female athymic nude mice (*n* = 6). Animals were monitored three times weekly for tumor formation, ascites development, and survival up to 40 days. At the end of the experiments animals were sacrificed and dissected. Two co-authors (H.G.M. and J.X.) conducted the animal experiments, but did not analyze the outcomes. The co-author (M.V.B.) who did not directly conduct animal experiments and did not have information on the conditions pertaining to each experimental group has analyzed the outcomes. Tumors formed in the abdominal region were collected, paraffin-embedded, and analyzed with hematoxylin & eosin staining as we described before^11,49,50^.

### Lipid uptake

Lipid uptake was assessed using two methods. QBT FA uptake assay was used as suggested by the manufacturer; fluorescence signal was detected using GloMax Multi Detection System (Promega) and plotted. FA uptake was also monitored by incubating cells in the presence of 500 μM linoleic acid for 24 h, fixing with 4% PFA, and staining with Oil red O solution for 30 min followed by red fluorescence imaging.

### Statistics

For analysis of survival a non-parametric test, the log-rank (Mantel−Cox) test, was employed using cBioportal and KM Plotter software. Comet assay parameters, including percent of tail DNA, tail length, tail moment, and olive tail moment, were analyzed using one-way ANOVA and MANOVA and GraphPad Prism software. Comparisons between two datasets with normal distribution were conducted using Student’s *t* test and Microsoft Excel software. A series of non-parametric tests, including Tukey HSD, Scheffe, Bonferroni and Holm, and Mann−Whitney *U* tests were used to compare two datasets with abnormal distribution. Data belonging to three or more independent groups were analyzed using one-way ANOVA and GraphPad Prism software. The findings were considered statistically significant at *p* < 0.05.

## Electronic supplementary material

supplementary figure legends

supplementary results

supplementary figure 1

supplementary figure 2

supplementary figure 3

supplementary figure 4

supplementary figure 5

supplementary figure 6

supplementary figure 7

supplementary figure 8

supplementary figure 9

supplementary figure 10

## References

[1] Siegel RL, Miller KD, Jemal A (2017). Cancer statistics, 2017. CA: Cancer J. Clin..

[2] Matulonis UA (2016). Ovarian cancer. Nat. Rev. Dis. Prim..

[3] Terricabras E, Benjamim C, Godessart N (2004). Drug discovery and chemokine receptor antagonists: eppur si muove!. Autoimmun. Rev..

[4] De Clercq E (2003). The bicyclam AMD3100 story. Nat. Rev. Drug Discov..

[5] Ridderstad Wollberg A (2014). Pharmacological inhibition of the chemokine receptor CX3CR1 attenuates disease in a chronic-relapsing rat model for multiple sclerosis. Proc. Natl. Acad. Sci. USA.

[6] Muralidhar GG, Barbolina MV (2015). The miR-200 family: versatile players in epithelial ovarian cancer. Int. J. Mol. Sci..

[7] Tsou CL, Haskell CA, Charo IF (2001). Tumor necrosis factor-alpha-converting enzyme mediates the inducible cleavage of fractalkine. J. Biol. Chem..

[8] Imai T (1997). Identification and molecular characterization of fractalkine receptor CX3CR1, which mediates both leukocyte migration and adhesion. Cell.

[9] Kim M, Rooper L, Xie J, Kajdacsy-Balla AA, Barbolina MV (2012). Fractalkine receptor CX(3)CR1 is expressed in epithelial ovarian carcinoma cells and required for motility and adhesion to peritoneal mesothelial cells. Mol. Cancer Res..

[10] Gaudin F (2011). Identification of the chemokine CX3CL1 as a new regulator of malignant cell proliferation in epithelial ovarian cancer. PLoS ONE.

[11] Gurler Main H (2016). Emergent role of the fractalkine axis in dissemination of peritoneal metastasis from epithelial ovarian carcinoma. Oncogene.

[12] Gyorffy B, Lanczky A, Szallasi Z (2012). Implementing an online tool for genome-wide validation of survival-associated biomarkers in ovarian-cancer using microarray data from 1287 patients. Endocr. Relat. Cancer.

[13] Penzvalto Z (2014). MEK1 is associated with carboplatin resistance and is a prognostic biomarker in epithelial ovarian cancer. Bmc Cancer.

[14] Ewald B, Sampath D, Plunkett W (2008). Nucleoside analogs: molecular mechanisms signaling cell death. Oncogene.

[15] Frankenberg-Schwager M (2005). Cisplatin-mediated DNA double-strand breaks in replicating but not in quiescent cells of the yeast Saccharomyces cerevisiae. Toxicology.

[16] Nowosielska A, Marinus MG (2005). Cisplatin induces DNA double-strand break formation in Escherichia coli dam mutants. Dna Repair. (Amst.)..

[17] Tomicic MT, Kaina B (2013). Topoisomerase degradation, DSB repair, p53 and IAPs in cancer cell resistance to camptothecin-like topoisomerase I inhibitors. Biochim. Biophys. Acta.

[18] Anglesio MS (2013). Type-specific cell line models for type-specific ovarian cancer research. PLoS ONE.

[19] Domcke S, Sinha R, Levine DA, Sander C, Schultz N (2013). Evaluating cell lines as tumour models by comparison of genomic profiles. Nat. Commun..

[20] Kuo LJ, Yang LX (2008). Gamma-H2AX—a novel biomarker for DNA double-strand breaks. Vivo.

[21] Banath JP, Klokov D, MacPhail SH, Banuelos CA, Olive PL (2010). Residual gammaH2AX foci as an indication of lethal DNA lesions. Bmc Cancer.

[22] Acosta JC (2008). Chemokine signaling via the CXCR2 receptor reinforces senescence. Cell.

[23] Cahill D, Connor B, Carney JP (2006). Mechanisms of eukaryotic DNA double strand break repair. Front. Biosci..

[24] Shrivastav M, De Haro LP, Nickoloff JA (2008). Regulation of DNA double-strand break repair pathway choice. Cell Res..

[25] Sonoda E, Hochegger H, Saberi A, Taniguchi Y, Takeda S (2006). Differential usage of non-homologous end-joining and homologous recombination in double strand break repair. Dna Repair. (Amst.)..

[26] Czornak K, Chughtai S, Chrzanowska KH (2008). Mystery of DNA repair: the role of the MRN complex and ATM kinase in DNA damage repair. J. Appl. Genet..

[27] van den Bosch M, Bree RT, Lowndes NF (2003). The MRN complex: coordinating and mediating the response to broken chromosomes. Embo Rep..

[28] Luoto KR (2010). Tumor cell kill by c-MYC depletion: role of MYC-regulated genes that control DNA double-strand break repair. Cancer Res..

[29] Petroni M (2016). The MRN complex is transcriptionally regulated by MYCN during neural cell proliferation to control replication stress. Cell Death Differ..

[30] Wang W (2016). MEF2C protects bone marrow B-lymphoid progenitors during stress haematopoiesis. Nat. Commun..

[31] Watson JV, Curling OM, Munn CF, Hudson CN (1987). Oncogene expression in ovarian cancer: a pilot study of c-myc oncoprotein in serous papillary ovarian cancer. Gynecol. Oncol..

[32] Cerami E (2012). The cBio cancer genomics portal: an open platform for exploring multidimensional cancer genomics data. Cancer Discov..

[33] Gao J (2013). Integrative analysis of complex cancer genomics and clinical profiles using the cBioPortal. Sci. Signal..

[34] Reyes-Gonzalez JM (2015). Targeting c-MYC in platinum-resistant ovarian cancer. Mol. Cancer Ther..

[35] Goldoni M, Johansson C (2007). A mathematical approach to study combined effects of toxicants in vitro: evaluation of the Bliss independence criterion and the Loewe additivity model. Toxicol. In. Vitr..

[36] Lengyel E (2010). Ovarian cancer development and metastasis. Am. J. Pathol..

[37] Tan DS, Agarwal R, Kaye SB (2006). Mechanisms of transcoelomic metastasis in ovarian cancer. Lancet Oncol..

[38] Nieman KM (2011). Adipocytes promote ovarian cancer metastasis and provide energy for rapid tumor growth. Nat. Med..

[39] Im DS (2013). Intercellular lipid mediators and GPCR drug discovery. Biomol. Ther. (Seoul).

[40] Im DS, Heise CE, Nguyen T, O’Dowd BF, Lynch KR (2001). Identification of a molecular target of psychosine and its role in globoid cell formation. J. Cell. Biol..

[41] MacPhee CE, Hatters DM, Sawyer WH, Howlett GJ (2000). Apolipoprotein C-II39-62 activates lipoprotein lipase by direct lipid-independent binding. Biochemistry.

[42] Matsumoto A (1990). Human macrophage scavenger receptors: primary structure, expression, and localization in atherosclerotic lesions. Proc. Natl. Acad. Sci. USA.

[43] Paulusma CC, Elferink RP (2010). P4 ATPases—the physiological relevance of lipid flipping transporters. FEBS Lett..

[44] Rohwedder A, Zhang Q, Rudge SA, Wakelam MJ (2014). Lipid droplet formation in response to oleic acid in Huh-7 cells is mediated by the fatty acid receptor FFAR4. J. Cell. Sci..

[45] Sanchez-Barrena MJ (2012). Bin2 is a membrane sculpting N-BAR protein that influences leucocyte podosomes, motility and phagocytosis. PLoS ONE.

[46] Sawamura T (1997). An endothelial receptor for oxidized low-density lipoprotein. Nature.

[47] Wang J, Wu X, Simonavicius N, Tian H, Ling L (2006). Medium-chain fatty acids as ligands for orphan G protein-coupled receptor GPR84. J. Biol. Chem..

[48] Brenner DJ (2008). The linear-quadratic model is an appropriate methodology for determining isoeffective doses at large doses per fraction. Semin. Radiat. Oncol..

[49] Desjardins M (2014). Versican regulates metastasis of epithelial ovarian carcinoma cells and spheroids. J. Ovarian Res..

[50] Kim M (2012). The lymphotactin receptor is expressed in epithelial ovarian carcinoma and contributes to cell migration and proliferation. Mol. Cancer Res..

