# Supplementary material for: Regulation of DNA damage repair and lipid uptake by CX3CR1 in epithelial ovarian carcinoma
Source: Oncogenesis. 2018 May 1;7(5):37. doi: 10.1038/s41389-018-0046-6 (PMC5928120; doi:10.1038/s41389-018-0046-6)
Supplement: Supplementary file 1 — supplementary figure legends [file 41389_2018_46_MOESM1_ESM.docx]

**SUPPLEMENTARY FIGURE LEGENDS**

**SUPPLEMENTARY FIGURE 1.** **Downregulation of CX_3_CR1 synergizes with x-ray radiation to reduce clone formation.**  Cell lines Caov-3 and SKOV-3 were transiently transfected with either CX_3_CR1-specific (designated “CX_3_CR1si”), control (designated “Ctrlsi”), or vehicle (designated “NT”). Caov-3 were subjected to 0, 1, 2, and 3 gray x-ray radiation on the 3^rd^ day following transfection. SKOV-3 cultured in monolayers were subjected to 0, 3, and 7 gray x-ray radiation on the 3^rd^ day following transfection. To assess radiosensitivity in spheroids, SKOV-3 were cultured on non-adhesive agarose gels two days prior to irradiation to create spheroids. One percent of cells was seeded to examine clone formation for 10 days followed by cell fixation and staining with crystal violet and imaging with BIO-RAD Chemidoc software. Surviving fraction of cells was found by detecting OD450 values and plotting using SigmaPlot software. CX_3_CR1 expression was determined by Western blot; β-actin was used as loading control, and normalized expression of CX_3_CR1 was calculated using digital densitometry. ***p*<0.01, two-way ANOVA test.

**SUPPLEMENTARY FIGURE 2.** **Downregulation CX_3_CR1 induces formation of persistent DSBs in irradiated cells**. Caov-3 was transiently transfected with either CX_3_CR1-specific (designated “CX_3_CR1si”), control (designated “Ctrlsi”) or vehicle (designated “NT”), subjected to 0 or 1 gray x-ray radiation on the 3^rd^ day following transfection. Cells were plated on glass cover slips either 20 min or 24 h after irradiation, as indicated, and stained with γH2AX-specific antibodies. Nuclear DNA was stained with DAPI. Images were superimposed from red and blue channels. Number of γH2AX-positive foci (red fluorescence) was quantified using Zeiss Axiovert software and plotted. **p*<0.05, Student’s t-test. Bar, 25 micron. Nuclei outlined with dotted lines were enlarged and shown at corners of the corresponding images.

**SUPPLEMENTARY FIGURE 3. Combination of CX_3_CR1 downregulation and x-ray radiation induces double strand DNA breaks**. SKOV-3 was transiently transfected with either CX_3_CR1-specific (designated “CX_3_CR1si”), control (designated “Ctrlsi”) or vehicle (designated “NT”), and subjected to 0 or 3 gray x-ray radiation on the 3^rd^ day following transfection. (**A**) Cells were plated on glass cover slips 24 h after irradiation, as indicated, and stained with γH2AX-specific antibodies. Nuclear DNA was stained with DAPI. Images were superimposed from green and blue channels. Bar, 25 micron. Nuclei outlined with dotted lines were enlarged and shown at corners of the corresponding images. Expression of γH2AX was examined with Western blot; TUBB served as loading control. (**B**) Neutral comet assay was performed as detailed in Methods. Mean tail DNA (%), mean tail length, mean tail moment, and mean olive tail moment were quantified as an average from three independent experiments, plotted and tabulated. **p*<0.05, one-way ANOVA and MANOVA tests. Immunofluorescence images (in black&white) show typical nuclei of cells in all tested conditions subjected to comet assay. Bar, 10 micron.

**SUPPLEMENTARY FIGURE 4. Downregulation of CX_3_CR1 reduces activation of DNA damage repair-related kinases in irradiated cells.** (**A**) Caov-3 was transiently transfected with either CX_3_CR1-specific (designated “CX_3_CR1si”), control (designated “Ctrlsi”) or vehicle (designated “NT”), and subjected to 0 or 1 gray x-ray radiation on the 3^rd^ day following transfection. Cells were seeded on glass coverslips. Expression of phosphorylated ATM (at Ser1981) was examined with immunofluorescence staining. Nuclear DNA was stained using DAPI. Nuclei outlined with dotted lines were enlarged and shown at the upper left corners of the corresponding images. Images shown were superimposed from blue and green channels. Number of pATM-positive foci (green fluorescence) in three independent experiments was quantified using Zeiss Axiovert software, averaged, and plotted. ***p*<0.01, Student’s t-test. Bar, 25 micron. Caov-3 (**B**) and SKOV-3 (**C**) were transiently transfected with either CX_3_CR1-specific (designated “CX_3_CR1si”), control (designated “Ctrlsi”) or vehicle (designated “NT”), and irradiated, or not, on the 3^rd^ day following transfection. Expression of phospho-ATM (at Ser1981), total ATM, phospho-CHEK1 (at Ser317), total CHEK1, phospho-CHEK2 (at Thr68), total CHEK2, phospho-PRKDC (at Thr2609), and total PRKDC were examined with Western blot and quantified using digital densitometry; TUBB or TUBA1A were used as loading controls.

**SUPPLEMENTARY FIGURE 5. An inhibitor of ATM, Ku55933, further radiosensitizes CX_3_CR1-deficient cells and further reduces activation of ATM in irradiated cells.** (**A**) Caov-3 was transiently transfected with either CX_3_CR1-specific (designated “CX_3_CR1si”), control (designated “Ctrlsi”) or vehicle (designated “NT”), treated with either 5 μM KU55933 or DMSO for 30 min prior to irradiation, and subjected to 0 or 1 gray x-ray radiation on the 3^rd^ day following transfection. One percent of cells was seeded to examine clone formation for 10 days followed by cell fixation, staining with crystal violet, and imaging with BIO-RAD Chemidoc software. Surviving fraction of cells was found by detecting OD450 values and plotting using SigmaPlot software. Radiosensitization shown in the table was determined if both SF2_control_/SF2_CX3CR1_ and D10_control_/D10_CX3CR1_ were >1.1. (**B**) Expression of phospho-ATM (Ser1981) and total ATM was examined with Western blot; TUBB was used as loading control. Normalized expression of phospho-ATM in three independent experiments was quantified with digital densitometry, averaged, and plotted. **p*<0.05, Student’s t-test.

**SUPPLEMENTARY FIGURE 6. MYCN is neither regulated by CX_3_CR1 or correlates with platinum response status and RAD50 expression in serous ovarian cystadenocarcinoma specimens.** (**A**) Coexpression of CX_3_CR1 and MYCN mRNAs in specimens of serous ovarian cystadenocarcinoma was analyzed using TCGA, Nature, 2011, dataset (n=557) and cBioportal software. Statistical parameters, including Pearson and Spearman correlation coefficients and *p*-values, are indicated on the graphs. (**B**) Expression of MYCN in OVCAR-4 transiently transfected with either CX_3_CR1-specific (designated “CX_3_CR1si”) or control (designated “Ctrl si”) or vehicle (designated “NT”) was examined using Western blot; ACTB served as a loading control. MYCN expression was quantified with digital densitometry. (**C**) Correlation between MYCN mRNA expression and platinum response status in specimens of serous ovarian cystadenocarcinoma was analyzed using TCGA, Nature, 2011, dataset (n=557) and cBioportal software. The data were statistically analyzed with Mann Whitney U test. (**D**) Coexpression of MYCN mRNA with that of MRE11A, RAD50, and NBN in specimens of serous ovarian cystadenocarcinoma was analyzed using TCGA, Nature, 2011, dataset (n=557) and cBioportal software. Statistical parameters, including Pearson and Spearman correlation coefficients and *p*-values, are indicated on the graphs.

**SUPPLEMENTARY FIGURE 7. MEF2C is regulated by CX_3_CR1 and correlates with invasion and shorter survival, but not with expression of MRN components or platinum status.** (**A**) Coexpression of CX_3_CR1 and MEF2C mRNAs in specimens of serous ovarian cystadenocarcinoma was analyzed using TCGA, Nature, 2011, dataset (n=557) and cBioportal software. Statistical parameters, including Pearson and Spearman correlation coefficients and *p*-values, are indicated on the graphs. (**B**) Specimens with serous ovarian cystadenocarcinoma (n=603, TCGA, Provisional dataset) were analyzed to identify correlation between MEF2C protein expression and vascular and lymphovascular invasion. Data were statistically analyzed with Mann Whitney U test and *p* values are shown on the graphs. (**C**) Overall survival of patients with MEF2C upregulation (red line) and no changes in MEF2C expression (blue line) according to the TCGA, Nature, 2011 (n=557), dataset was plotted and analyzed with Kaplan-Meier plot using cBioportal software and Log-rank (Mantel-Cox) test. Median survival and the number of cases are indicated in the table. (**D**) Expression of MEF2C in SKOV-3 and OVCAR-4 transiently transfected with either CX_3_CR1-specific (designated “CX_3_CR1si”) or control (designated “Ctrl si”) or vehicle (designated “NT”) was examined using Western blot (shown, OVCAR-4); ACTB served as a loading control. MEF2C expression was quantified with digital densitometry. Coexpression of MEF2C mRNA and MRE11A mRNA and protein (**E**) and coexpression of MEF2C mRNA and RAD50 mRNA and protein (**F**) in specimens of serous ovarian cystadenocarcinoma was analyzed using TCGA, Provisional, dataset (n=603) and cBioportal software. Statistical parameters, including Pearson and Spearman correlation coefficients and *p*-values are indicated on the graphs. (**G**) Correlation between MEF2C mRNA expression and platinum response status in specimens of serous ovarian cystadenocarcinoma was analyzed using TCGA, Nature, 2011, dataset (n=557) and cBioportal software. The data were statistically analyzed with Mann Whitney U test.

**SUPPLEMENTARY FIGURE 8. Combination of CX_3_CR1 downregulation and platinum-based drugs significantly and synergistically reduces clone formation and induces double strand DNA breaks**. OVCAR-4 was transiently transfected with either CX_3_CR1-specific (designated “CX_3_CR1si”) or control (designated “Ctrl si”) and subjected to cisplatin at indicated concentrations for 24 h on the 3^rd^ day post transfection. (**A**) Cytotoxicity of cisplatin treatment was determined using WST-1 assay at the end of cisplatin treatment, and the average of three independent experiments is plotted. Clone formation in cells treated with cisplatin was determined as described in Methods, and the average of three independent experiments is plotted. **p*<0.05, Student’s t-test. Percent of the effect was determined based on the clonogenic assay and calculated as (1 – [surviving fraction])×100. The gray dashed line represents a threshold above which the effect of the combined treatment is synergistic, or below which the effect is antagonistic. (**B**) Cells were plated on glass cover slips 24 h after cisplatin treatment and stained with γH2AX-specific antibodies. Nuclear DNA was stained with DAPI. Images were superimposed from green and blue channels. Number of γH2AX-positive foci (green fluorescence) from three independent experiments was quantified using Zeiss Axiovert software and plotted. **p*<0.05, Student’s t-test. Bar, 30 micron. Nuclei outlined with dotted lines were enlarged and shown at corners of the corresponding images. (**C**) Neutral comet assay was performed as detailed in Methods. Mean tail DNA (%), mean tail length, mean tail moment, and mean olive tail moment were quantified as an average from three independent experiments, plotted, and tabulated. **p*<0.05, one-way ANOVA and MANOVA tests. Immunofluorescence images (in black&white) show typical nuclei of cells in all tested conditions subjected to comet assay. Bar, 10 micron. (**D**) Cytotoxicity of carboplatin treatment was determined using WST-1 assay at the end of carboplatin treatment, and the average of three independent experiments is plotted. Percent of the effect was calculated as (1 – [surviving fraction])×100. The gray dashed line represents a threshold above which the effect of the combined treatment is synergistic, or below which the effect is antagonistic. (**E**) Clone formation in cells treated with carboplatin was determined as described in Methods, and the average of three independent experiments is plotted. **p*<0.05, Student’s t-test. Percent of the effect was calculated as (1 – [surviving fraction])×100. The gray dashed line represents a threshold above which the effect of the combined treatment is synergistic, or below which the effect is antagonistic. Combination Index (CI) values are shown on the graphs.

**SUPPLEMENTARY FIGURE 9. High CX_3_CR1 expression predicts shorter post-progression survival (PPS) of serous ovarian carcinoma patients treated with platinum therapies, gemcitabine, and topotecan**. PPS of serous ovarian carcinoma patients treated with platinum therapies, gemcitabine, and topotecan as a function of CX_3_CR1 expression were analyzed using KM Plotter database. Red lines – high CX_3_CR1, black lines – low CX_3_CR1. Numbers of specimens with high (red) and low (black) CX_3_CR1 for each analyzed group and their corresponding average survival are shown in the tables. Expression of CX_3_CR1 in examined specimens is plotted as beeswarm plots shown as inserts; red – high CX_3_CR1, black – low CX_3_CR1. Survival was analyzed with Mantel-Cox log-rank test using KM Plotter software; hazard ratios (HR) and *p*-values are indicated on the graphs.

**SUPPLEMENTARY FIGURE 10. Expression of GPR65 and FFAR4 correlates with progression-free survival in serous ovarian carcinoma and is regulated by CX_3_CR1**. PFS of serous ovarian carcinoma patients as a function of high and low GPR65 (**A**) and FFAR4 (**B**) expression were analyzed using KM Plotter database. Red lines – high GPR65 (or FFAR4), black lines – low GPR65 (or FFAR4). Numbers of specimens with high (red) and low (black) GPR65 (or FFAR4) for each analyzed group and their corresponding average survival are shown in the tables. The best preforming threshold was used to determine “low” and “high” groups of specimens using KM Plotter software. Expression of GPR65 (or FFAR4) in examined specimens is plotted as beeswarm plots shown as inserts; red – high GPR65 (or FFAR4), black – low GPR65 (or FFAR4). Survival was analyzed with Mantel-Cox log-rank test using KM Plotter software; hazard ratios (HR) and *p*-values are indicated on the graphs.
