# Supplementary material for: Regulation of DNA damage repair and lipid uptake by CX3CR1 in epithelial ovarian carcinoma
Source: Oncogenesis. 2018 May 1;7(5):37. doi: 10.1038/s41389-018-0046-6 (PMC5928120; doi:10.1038/s41389-018-0046-6)
Supplement: Supplementary file 2 — supplementary results [file 41389_2018_46_MOESM2_ESM.docx]

**SUPPLEMENTARY RESULTS**

A recent study identified MYCN as a regulator of expression of MRN components in neural progenitor cells (1). However, our analysis of CX_3_CR1 and MYCN mRNA co-expression in serous cystadenocarcinoma specimens demonstrated moderate inverse correlation, and Western blot revealed no changes in MYCN expression when CX_3_CR1 was downregulated with siRNAs (Supplementary Figure 6A,B), suggesting that MYCN is not involved in regulation of CX_3_CR1-dependent expression of MRN complex proteins expression in ovarian carcinoma, because MYCN expression does not depend on CX_3_CR1. Additionally, expression of MYCN did not correlate with platinum status (Supplementary Figure 6C) and its mRNA expression did not affect expression of either MRE11A, RAD50, or NBN mRNAs in specimens of serous ovarian cystadenocarcinoma (Supplementary Figure 6D), further indicating that MYCN is not involved in CX_3_CR1-dependent DNA damage response in ovarian carcinoma.

It has been previously demonstrated that expression of the MRN complex proteins RAD50 and MRE11A is controlled by MEF2C in bone marrow B-cell progenitor cells (2). Analysis of co-expression of CX_3_CR1 mRNA with that of MEF2C in specimens of serous ovarian cystadenocarcinoma (TCGA, cBioportal) indicated that changes in mRNA expression for CX_3_CR1 and MEF2C statistically significantly coincide (Supplementary Figure 7A). Expression of MEF2C protein significantly correlated with vascular invasion as well as disease progression and recurrence in specimens of serous cystadenocarcinoma (Supplementary Figure 7B). Patients with upregulation of MEF2C survived significantly shorter than those without alterations in its expression (Supplementary Figure 7C). Altogether, these data suggest that upregulation of MEF2C is important for ovarian cancer progression. To determine the relationship between expression of CX_3_CR1 and MEF2C, we tested expression of MEF2C in cells with downregulated CX_3_CR1 by Western blot and found that it was strongly downregulated, suggesting a role of CX_3_CR1 in MEF2C expression (Supplementary Figure 7D). These data suggest existence of a common regulatory mechanism shared by multiple cancer types whereby CX_3_CR1 regulates expression of MEF2C. However, both mRNA and protein expression of MRE11A and RAD50 did not correlate with MEF2C mRNA expression (Supplementary Figure 7E,F), suggesting that MEF2C, although potentially important for ovarian carcinoma progression, is unlikely to regulate MRN proteins in a CX_3_CR1-dependent manner. Moreover, expression of MEF2C mRNA did not correlate with platinum status (Supplementary Figure 7G), suggesting that it does not play a role in DNA damage response.

**references**

1. Petroni M, Sardina F, Heil C, Sahun-Roncero M, Colicchia V, Veschi V, et al. The MRN complex is transcriptionally regulated by MYCN during neural cell proliferation to control replication stress. Cell Death Differ. 2016;23(2):197-206.

2. Wang W, Org T, Montel-Hagen A, Pioli PD, Duan D, Israely E, et al. MEF2C protects bone marrow B-lymphoid progenitors during stress haematopoiesis. Nature communications. 2016;7:12376.
