# Supplementary material for: Regulation of DNA damage repair and lipid uptake by CX3CR1 in epithelial ovarian carcinoma
Source: Oncogenesis. 2018 May 1;7(5):37. doi: 10.1038/s41389-018-0046-6 (PMC5928120; doi:10.1038/s41389-018-0046-6)
Supplement: Supplementary file 3 — supplementary figure 1 [file 41389_2018_46_MOESM3_ESM.pptx]

## Slide 1
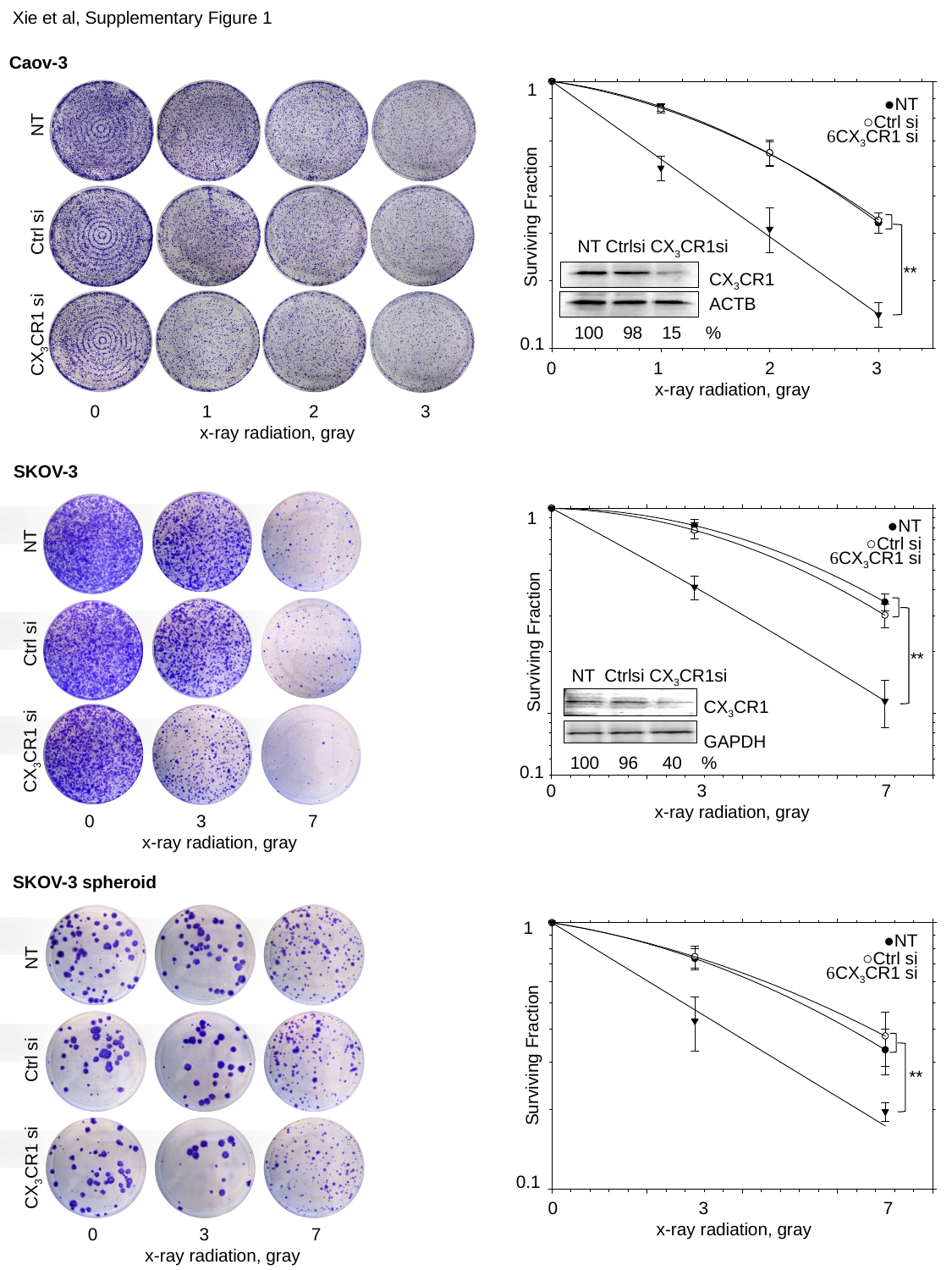

Xie et al, Supplementary Figure 1
Caov-3
CX3CR1 si Ctrl si NT
0 1 2 3
x-ray radiation, gray
1
0.1
●NT
○Ctrl si
CX3CR1 si
Surviving Fraction
NT Ctrlsi CX3CR1si
CX3CR1
ACTB
100 98 15 %
0 1 2 3
x-ray radiation, gray
**
SKOV-3
CX3CR1 si Ctrl si NT
0 3 7
x-ray radiation, gray
1
0.1
●NT
○Ctrl si
CX3CR1 si
Surviving Fraction
0 3 7
x-ray radiation, gray
**
NT Ctrlsi CX3CR1si
CX3CR1
GAPDH
100 96 40 %
SKOV-3 spheroid
CX3CR1 si Ctrl si NT
0 3 7
x-ray radiation, gray
1
0.1
●NT
○Ctrl si
CX3CR1 si
Surviving Fraction
0 3 7
x-ray radiation, gray
**
