# Supplementary material for: Regulation of DNA damage repair and lipid uptake by CX3CR1 in epithelial ovarian carcinoma
Source: Oncogenesis. 2018 May 1;7(5):37. doi: 10.1038/s41389-018-0046-6 (PMC5928120; doi:10.1038/s41389-018-0046-6)
Supplement: Supplementary file 4 — supplementary figure 2 [file 41389_2018_46_MOESM4_ESM.pptx]

## Slide 1
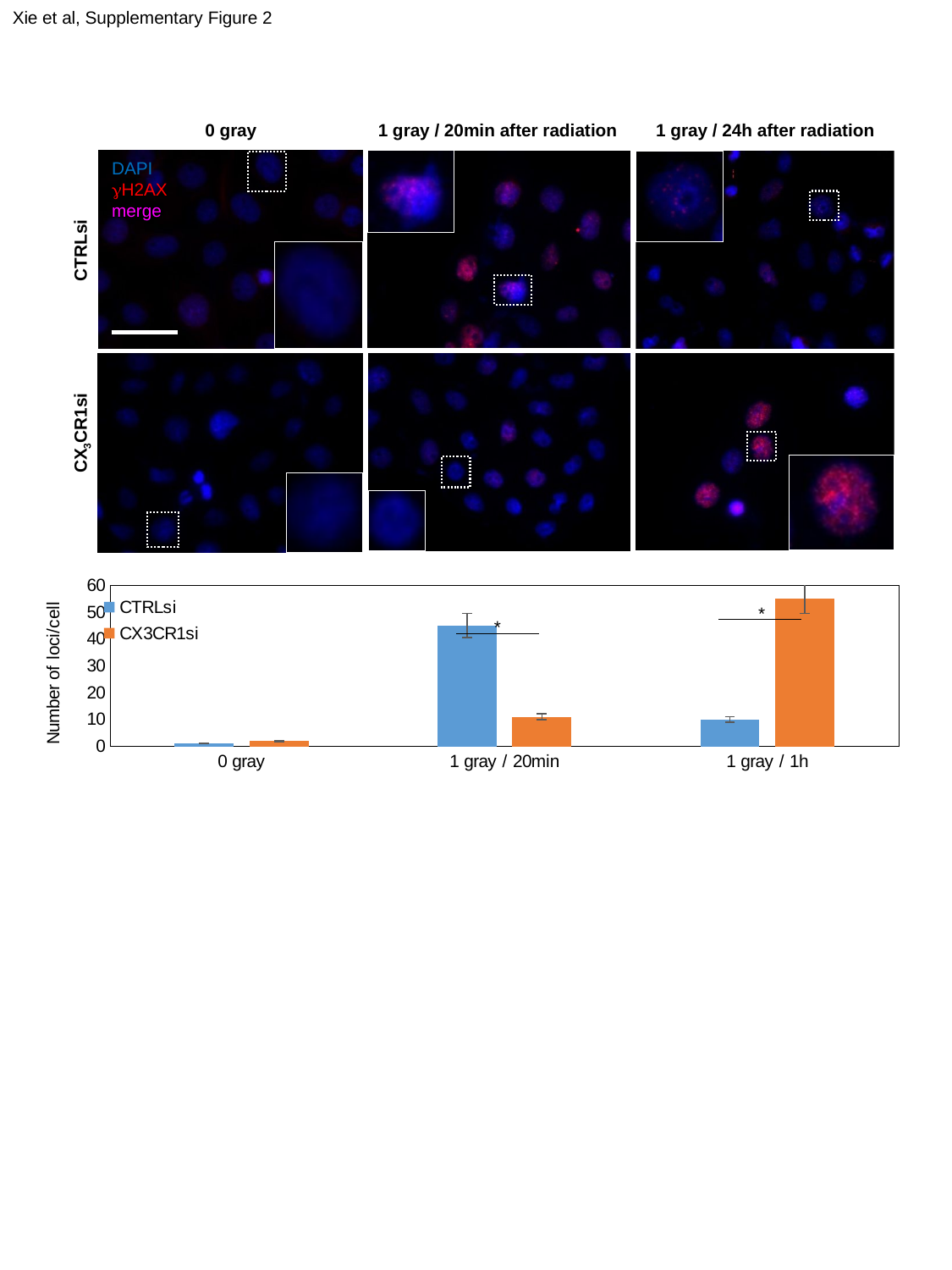

Xie et al, Supplementary Figure 2
0 gray 1 gray / 20min after radiation 1 gray / 24h after radiation
DAPI
γH2AX
merge
CX3CR1si CTRLsi
### Chart
| Category | CTRLsi | CX3CR1si |
|---|---|---|
| 0 gray | 1.0 | 2.0 |
| 1 gray / 20min | 45.0 | 11.0 |
| 1 gray / 1h | 10.0 | 55.0 |*
*
