# Supplementary material for: Regulation of DNA damage repair and lipid uptake by CX3CR1 in epithelial ovarian carcinoma
Source: Oncogenesis. 2018 May 1;7(5):37. doi: 10.1038/s41389-018-0046-6 (PMC5928120; doi:10.1038/s41389-018-0046-6)
Supplement: Supplementary file 5 — supplementary figure 3 [file 41389_2018_46_MOESM5_ESM.pptx]

## Slide 1
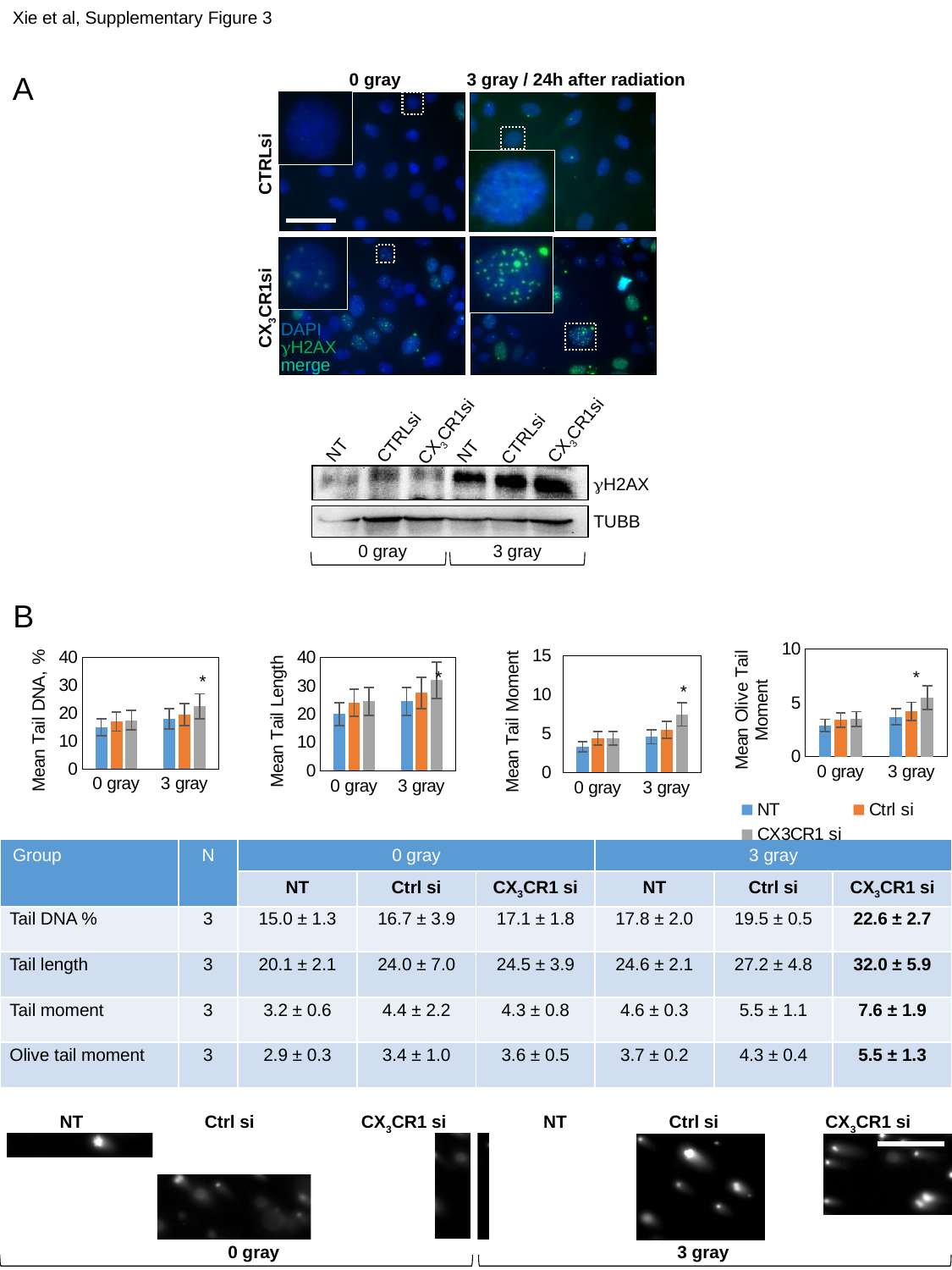

Xie et al, Supplementary Figure 3
0 gray
3 gray / 24h after radiation
CX3CR1si CTRLsi
DAPI
γH2AX
merge
A
CX3CR1si
CX3CR1si
CTRLsi
CTRLsi
NT
NT
γH2AX
TUBB
0 gray
3 gray
B
### Chart
| Category | NT | Ctrl si | CX3CR1 si |
|---|---|---|---|
| 0 gray | 2.9 | 3.4 | 3.5 |
| 3 gray | 3.7 | 4.2 | 5.5 |
### Chart
| Category | NT | Ctrl si | CX3CR1 si |
|---|---|---|---|
| 0 gray | 3.3 | 4.4 | 4.4 |
| 3 gray | 4.6 | 5.5 | 7.5 |
### Chart
| Category | NT | Ctrl si | CX3CR1 si |
|---|---|---|---|
| 0 gray | 15.0 | 17.0 | 17.5 |
| 3 gray | 18.0 | 19.5 | 22.5 |
### Chart
| Category | NT | Ctrl si | CX3CR1 si |
|---|---|---|---|
| 0 gray | 20.0 | 24.0 | 24.5 |
| 3 gray | 24.5 | 27.5 | 32.0 |*
*
*
*
| Group | N | 0 gray | | | 3 gray | | |
| --- | --- | --- | --- | --- | --- | --- | --- |
| | | NT | Ctrl si | CX3CR1 si | NT | Ctrl si | CX3CR1 si |
| Tail DNA % | 3 | 15.0 ± 1.3 | 16.7 ± 3.9 | 17.1 ± 1.8 | 17.8 ± 2.0 | 19.5 ± 0.5 | 22.6 ± 2.7 |
| Tail length | 3 | 20.1 ± 2.1 | 24.0 ± 7.0 | 24.5 ± 3.9 | 24.6 ± 2.1 | 27.2 ± 4.8 | 32.0 ± 5.9 |
| Tail moment | 3 | 3.2 ± 0.6 | 4.4 ± 2.2 | 4.3 ± 0.8 | 4.6 ± 0.3 | 5.5 ± 1.1 | 7.6 ± 1.9 |
| Olive tail moment | 3 | 2.9 ± 0.3 | 3.4 ± 1.0 | 3.6 ± 0.5 | 3.7 ± 0.2 | 4.3 ± 0.4 | 5.5 ± 1.3 |
NT Ctrl si CX3CR1 si NT Ctrl si CX3CR1 si
0 gray
3 gray
