# Supplementary material for: Regulation of DNA damage repair and lipid uptake by CX3CR1 in epithelial ovarian carcinoma
Source: Oncogenesis. 2018 May 1;7(5):37. doi: 10.1038/s41389-018-0046-6 (PMC5928120; doi:10.1038/s41389-018-0046-6)
Supplement: Supplementary file 6 — supplementary figure 4 [file 41389_2018_46_MOESM6_ESM.pptx]

## Slide 1
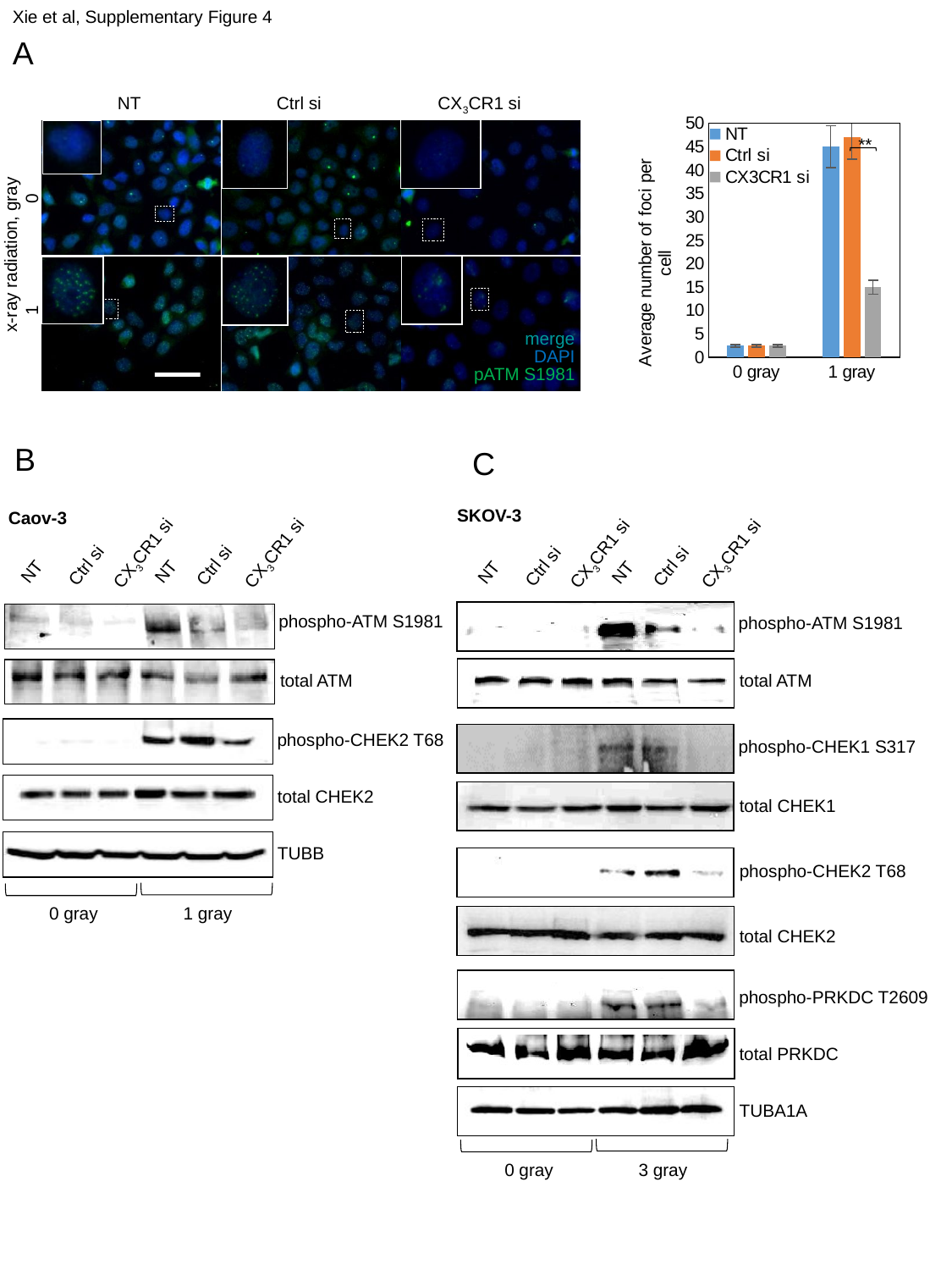

Xie et al, Supplementary Figure 4
A
NT Ctrl si CX3CR1 si
x-ray radiation, gray
1 0
### Chart
| Category | NT | Ctrl si | CX3CR1 si |
|---|---|---|---|
| 0 gray | 2.5 | 2.5 | 2.5 |
| 1 gray | 45.0 | 47.0 | 15.0 |
**
merge
DAPI
pATM S1981
NT
NT
NT
NT
Ctrl si
Ctrl si
SKOV-3
Ctrl si
Ctrl si
CX3CR1 si
CX3CR1 si
CX3CR1 si
CX3CR1 si
Caov-3
phospho-ATM S1981
phospho-ATM S1981
total ATM
total ATM
phospho-CHEK2 T68
phospho-CHEK1 S317
total CHEK2
total CHEK1
TUBB
phospho-CHEK2 T68
1 gray
0 gray
total CHEK2
phospho-PRKDC T2609
total PRKDC
TUBA1A
3 gray
0 gray
B
C
