# Supplementary material for: Regulation of DNA damage repair and lipid uptake by CX3CR1 in epithelial ovarian carcinoma
Source: Oncogenesis. 2018 May 1;7(5):37. doi: 10.1038/s41389-018-0046-6 (PMC5928120; doi:10.1038/s41389-018-0046-6)
Supplement: Supplementary file 7 — supplementary figure 5 [file 41389_2018_46_MOESM7_ESM.pptx]

## Slide 1
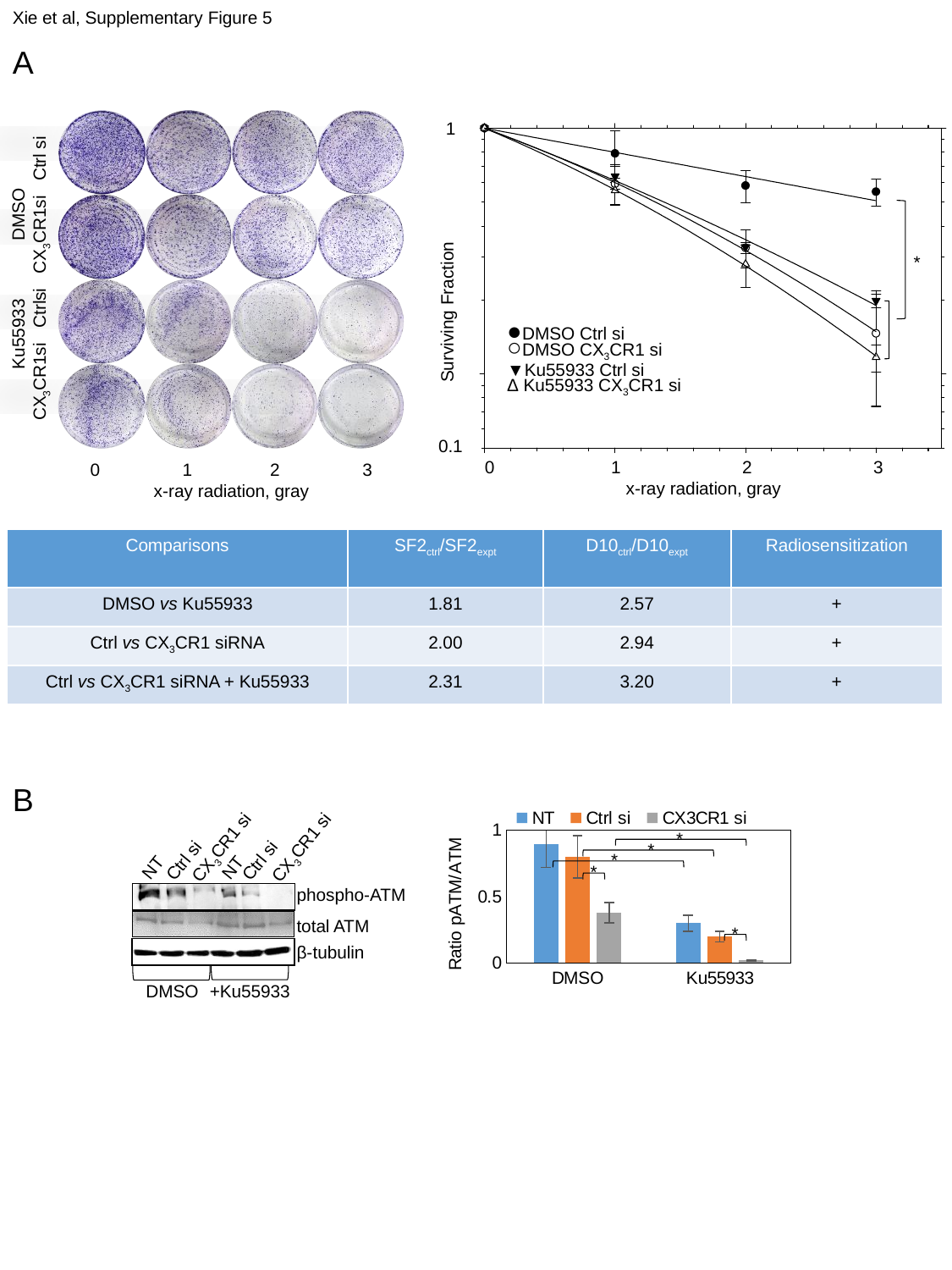

Xie et al, Supplementary Figure 5
Ku55933 DMSO
CX3CR1si Ctrlsi CX3CR1si Ctrl si
0 1 2 3
x-ray radiation, gray
A
1
0.1
*
Surviving Fraction
●DMSO Ctrl si
○DMSO CX3CR1 si
▼Ku55933 Ctrl si
Δ Ku55933 CX3CR1 si
0 1 2 3
 x-ray radiation, gray
| Comparisons | SF2ctrl/SF2expt | D10ctrl/D10expt | Radiosensitization |
| --- | --- | --- | --- |
| DMSO vs Ku55933 | 1.81 | 2.57 | + |
| Ctrl vs CX3CR1 siRNA | 2.00 | 2.94 | + |
| Ctrl vs CX3CR1 siRNA + Ku55933 | 2.31 | 3.20 | + |
B
### Chart
| Category | NT | Ctrl si | CX3CR1 si |
|---|---|---|---|
| DMSO | 0.9 | 0.8 | 0.38 |
| Ku55933 | 0.3 | 0.2 | 0.02 |
*
NT
NT
Ctrl si
Ctrl si
CX3CR1 si
CX3CR1 si
*
*
*
phospho-ATM
total ATM
*
β-tubulin
+Ku55933
DMSO
