# Supplementary material for: Regulation of DNA damage repair and lipid uptake by CX3CR1 in epithelial ovarian carcinoma
Source: Oncogenesis. 2018 May 1;7(5):37. doi: 10.1038/s41389-018-0046-6 (PMC5928120; doi:10.1038/s41389-018-0046-6)
Supplement: Supplementary file 8 — supplementary figure 6 [file 41389_2018_46_MOESM8_ESM.pptx]

## Slide 1
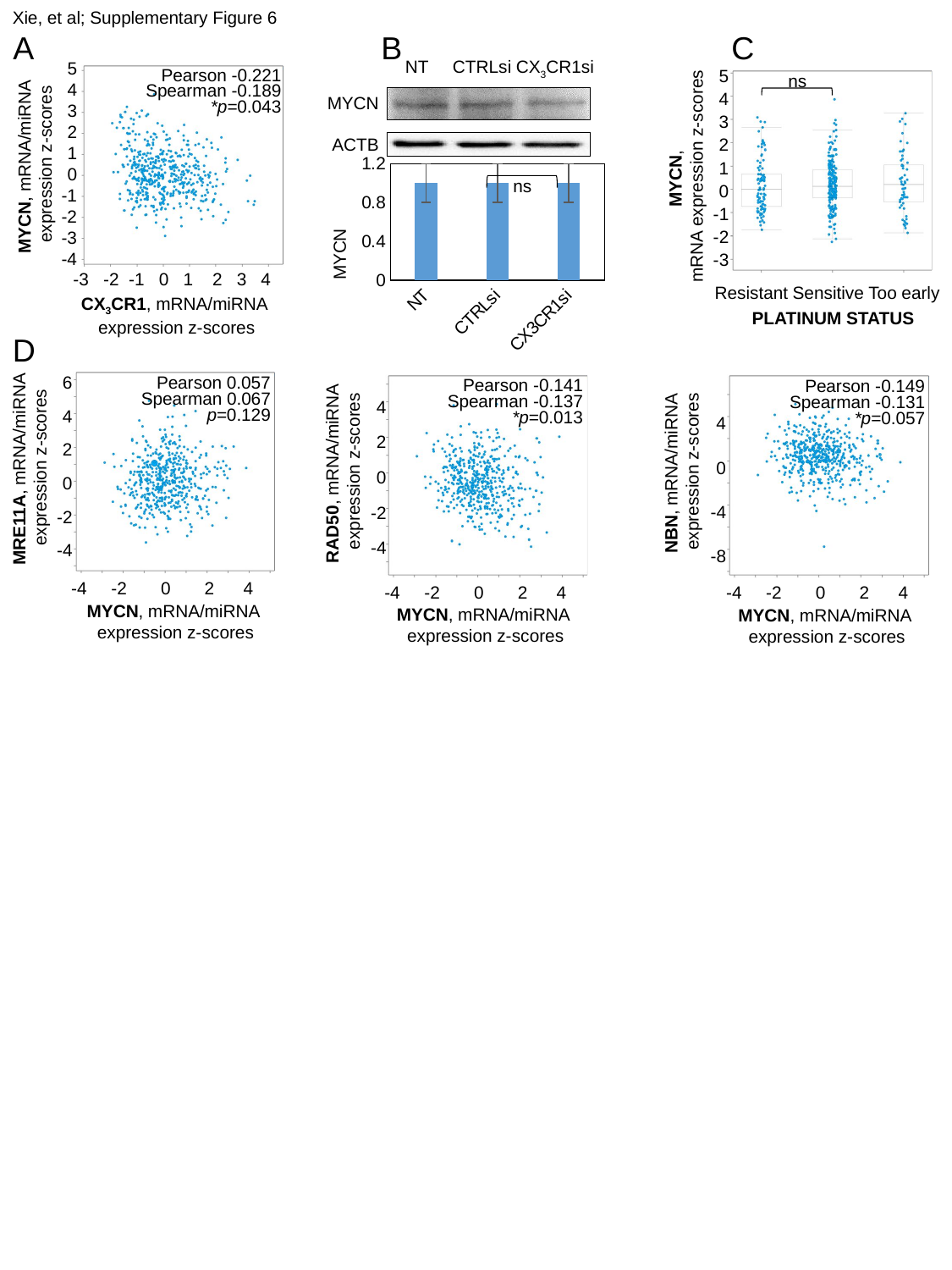

Xie, et al; Supplementary Figure 6
A
B
C
NT CTRLsi CX3CR1si
5
4
3
2
1
0
-1
-2
-3
-4
Pearson -0.221
Spearman -0.189
*p=0.043
MYCN, mRNA/miRNA
 expression z-scores
-3 -2 -1 0 1 2 3 4
CX3CR1, mRNA/miRNA
expression z-scores
5
4
3
2
1
0
-1
-2
-3
ns
MYCN,
mRNA expression z-scores
Resistant Sensitive Too early
PLATINUM STATUS
MYCN
ACTB
### Chart
| Category | mycn |
|---|---|
| NT | 1.0 |
| CTRLsi | 1.0 |
| CX3CR1si | 1.0 |ns
D
6
4
2
0
-2
-4
Pearson 0.057
Spearman 0.067
p=0.129
MRE11A, mRNA/miRNA
expression z-scores
-4 -2 0 2 4
MYCN, mRNA/miRNA
expression z-scores
Pearson -0.141
Spearman -0.137
*p=0.013
4
2
0
-2
-4
RAD50, mRNA/miRNA
expression z-scores
-4 -2 0 2 4
MYCN, mRNA/miRNA
expression z-scores
Pearson -0.149
Spearman -0.131
*p=0.057
4
0
-4
-8
NBN, mRNA/miRNA
expression z-scores
-4 -2 0 2 4
MYCN, mRNA/miRNA
expression z-scores
