# Supplementary material for: Regulation of DNA damage repair and lipid uptake by CX3CR1 in epithelial ovarian carcinoma
Source: Oncogenesis. 2018 May 1;7(5):37. doi: 10.1038/s41389-018-0046-6 (PMC5928120; doi:10.1038/s41389-018-0046-6)
Supplement: Supplementary file 9 — supplementary figure 7 [file 41389_2018_46_MOESM9_ESM.pptx]

## Slide 1
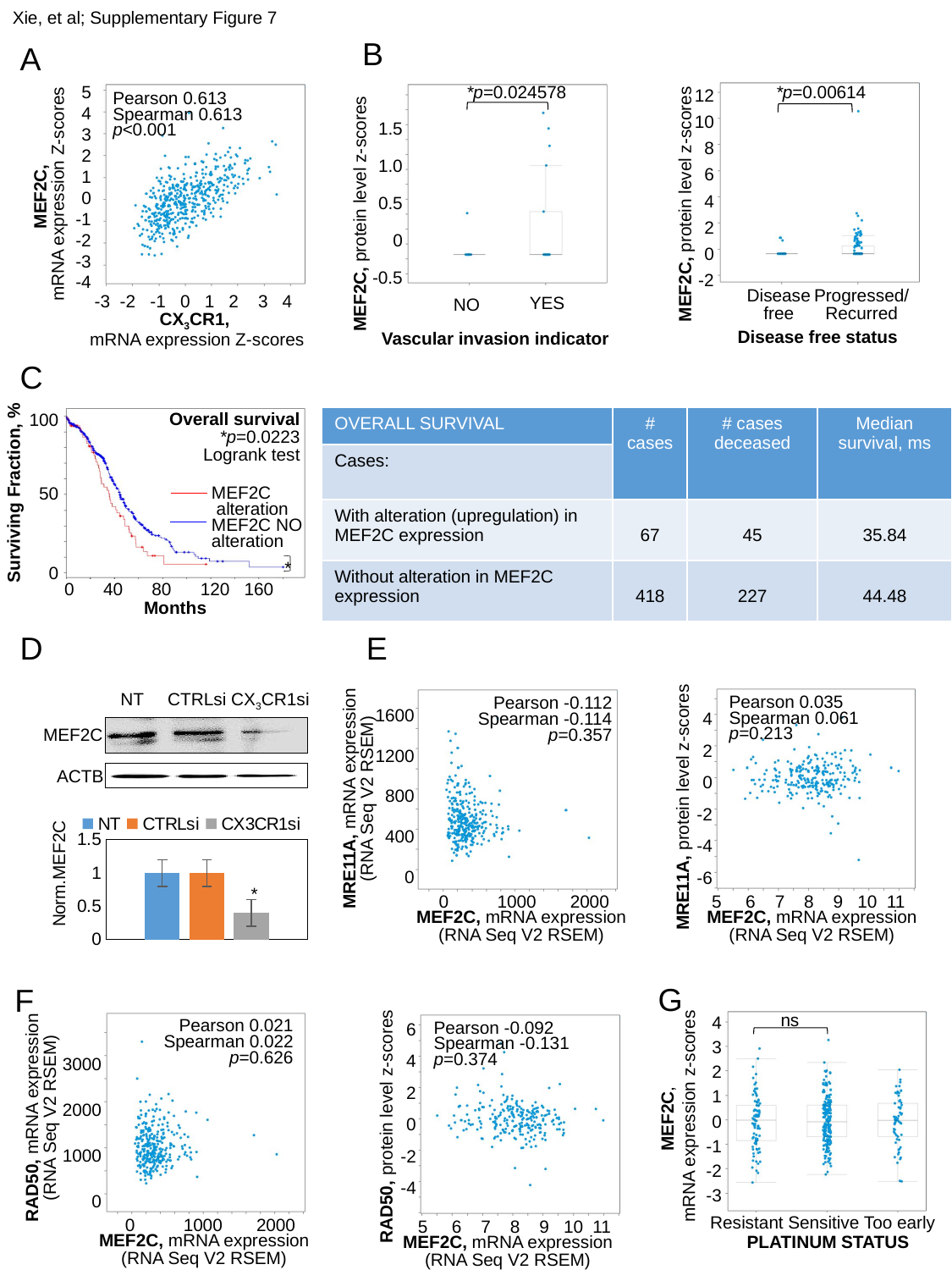

Xie, et al; Supplementary Figure 7
B
A
12
10
8
6
4
2
0
-2
*p=0.00614
MEF2C, protein level z-scores
Disease free
Progressed/
Recurred
Disease free status
5
4
3
2
1
0
-1
-2
-3
-4
Pearson 0.613
Spearman 0.613
p<0.001
MEF2C,
 mRNA expression Z-scores
-3 -2 -1 0 1 2 3 4
CX3CR1,
mRNA expression Z-scores
*p=0.024578
1.5
1.0
0.5
0
-0.5
MEF2C, protein level z-scores
YES
NO
Vascular invasion indicator
C
100
50
0
Overall survival
*p=0.0223
Logrank test
Surviving Fraction, %
MEF2C alteration
MEF2C NO alteration
*
0 40 80 120 160
Months
| OVERALL SURVIVAL | # cases | # cases deceased | Median survival, ms |
| --- | --- | --- | --- |
| Cases: | | | |
| With alteration (upregulation) in MEF2C expression | 67 | 45 | 35.84 |
| Without alteration in MEF2C expression | 418 | 227 | 44.48 |
E
D
Pearson 0.035
Spearman 0.061
p=0.213
4
2
0
-2
-4
-6
MRE11A, protein level z-scores
5 6 7 8 9 10 11
MEF2C, mRNA expression (RNA Seq V2 RSEM)
1600
1200
800
400
0
Pearson -0.112
Spearman -0.114
p=0.357
MRE11A, mRNA expression (RNA Seq V2 RSEM)
0 1000 2000
MEF2C, mRNA expression (RNA Seq V2 RSEM)
NT CTRLsi CX3CR1si
MEF2C
ACTB
### Chart
| Category | NT | CTRLsi | CX3CR1si |
|---|---|---|---|
| MEF2C | 1.0 | 1.0 | 0.4 |*
4
3
2
1
0
-1
-2
-3
ns
MEF2C,
mRNA expression z-scores
Resistant Sensitive Too early
PLATINUM STATUS
G
F
6
4
2
0
-2
-4
Pearson -0.092
Spearman -0.131
p=0.374
RAD50, protein level z-scores
5 6 7 8 9 10 11
MEF2C, mRNA expression (RNA Seq V2 RSEM)
Pearson 0.021
Spearman 0.022
p=0.626
3000
2000
1000
0
RAD50, mRNA expression (RNA Seq V2 RSEM)
0 1000 2000
MEF2C, mRNA expression (RNA Seq V2 RSEM)
