# Supplementary material for: Regulation of DNA damage repair and lipid uptake by CX3CR1 in epithelial ovarian carcinoma
Source: Oncogenesis. 2018 May 1;7(5):37. doi: 10.1038/s41389-018-0046-6 (PMC5928120; doi:10.1038/s41389-018-0046-6)
Supplement: Supplementary file 10 — supplementary figure 8 [file 41389_2018_46_MOESM10_ESM.pptx]

## Slide 1
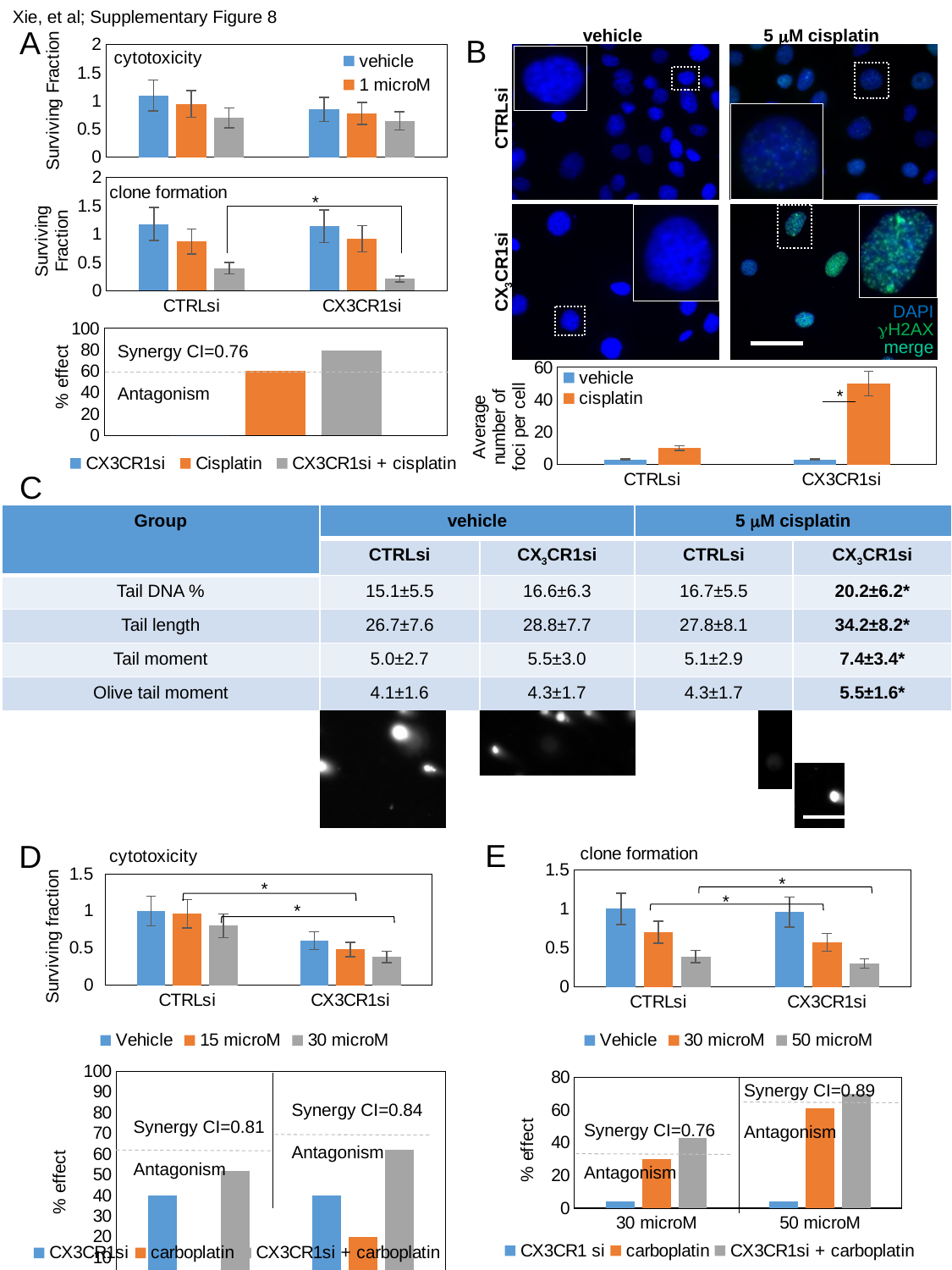

### Chart: cytotoxicity
| Category | vehicle | 1 microM | 5 microM |
|---|---|---|---|
| CTRLsi | 1.1 | 0.95 | 0.7 |
| CX3CR1si | 0.85 | 0.78 | 0.65 |Xie, et al; Supplementary Figure 8
A
vehicle 5 μM cisplatin
B
CX3CR1si CTRLsi
### Chart: clone formation
| Category | vehicle | 1 microM | 5 microM |
|---|---|---|---|
| CTRLsi | 1.18 | 0.87 | 0.4 |
| CX3CR1si | 1.14 | 0.92 | 0.21 |*
DAPI
γH2AX
merge
### Chart
| Category | CX3CR1si | Cisplatin | CX3CR1si + cisplatin |
|---|---|---|---|
| Series 1 | 0.0 | 60.0 | 79.0 |
Synergy CI=0.76
Antagonism
### Chart
| Category | vehicle | cisplatin |
|---|---|---|
| CTRLsi | 3.0 | 10.0 |
| CX3CR1si | 3.0 | 50.0 |*
C
| Group | vehicle | | 5 μM cisplatin | |
| --- | --- | --- | --- | --- |
| | CTRLsi | CX3CR1si | CTRLsi | CX3CR1si |
| Tail DNA % | 15.1±5.5 | 16.6±6.3 | 16.7±5.5 | 20.2±6.2\* |
| Tail length | 26.7±7.6 | 28.8±7.7 | 27.8±8.1 | 34.2±8.2\* |
| Tail moment | 5.0±2.7 | 5.5±3.0 | 5.1±2.9 | 7.4±3.4\* |
| Olive tail moment | 4.1±1.6 | 4.3±1.7 | 4.3±1.7 | 5.5±1.6\* |
### Chart: cytotoxicity
| Category | Vehicle | 15 microM | 30 microM |
|---|---|---|---|
| CTRLsi | 1.0 | 0.96 | 0.8 |
| CX3CR1si | 0.6 | 0.48 | 0.38 |
### Chart: clone formation
| Category | Vehicle | 30 microM | 50 microM |
|---|---|---|---|
| CTRLsi | 1.0 | 0.7 | 0.39 |
| CX3CR1si | 0.96 | 0.57 | 0.3 |*
*
E
D
*
*
### Chart
| Category | CX3CR1si | carboplatin | CX3CR1si + carboplatin |
|---|---|---|---|
| 15 microM | 40.0 | 4.0 | 52.0 |
| 30 microM | 40.0 | 20.0 | 62.0 |Synergy CI=0.84
Antagonism
Synergy CI=0.81
Antagonism
### Chart
| Category | CX3CR1 si | carboplatin | CX3CR1si + carboplatin |
|---|---|---|---|
| 30 microM | 4.0 | 30.0 | 43.0 |
| 50 microM | 4.0 | 61.0 | 70.0 |Synergy CI=0.89
Antagonism
Synergy CI=0.76
Antagonism
