# Supplementary material for: Regulation of DNA damage repair and lipid uptake by CX3CR1 in epithelial ovarian carcinoma
Source: Oncogenesis. 2018 May 1;7(5):37. doi: 10.1038/s41389-018-0046-6 (PMC5928120; doi:10.1038/s41389-018-0046-6)
Supplement: Supplementary file 11 — supplementary figure 9 [file 41389_2018_46_MOESM11_ESM.pptx]

## Slide 1
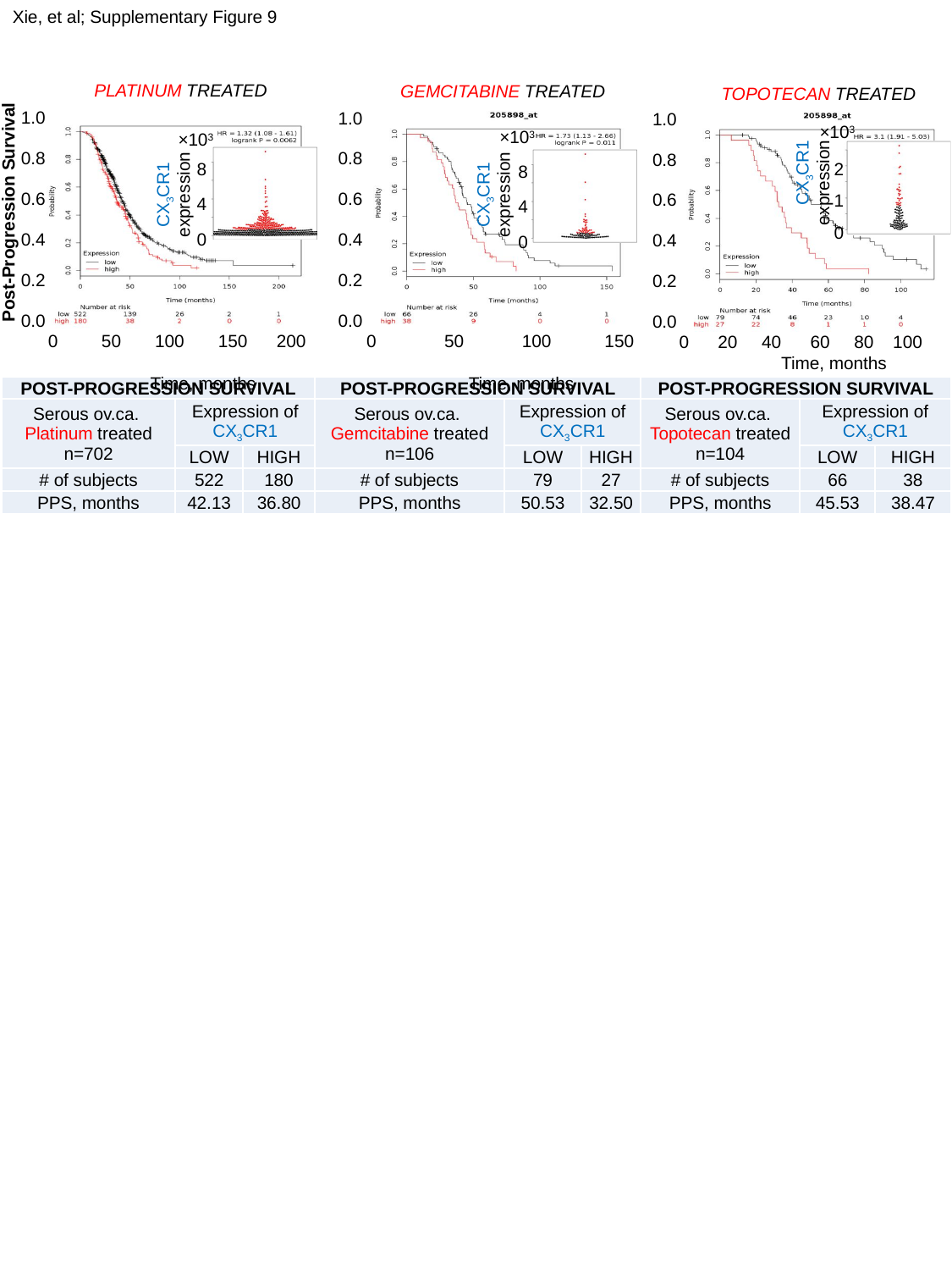

Xie, et al; Supplementary Figure 9
TOPOTECAN TREATED
1.0
0.8
0.6
0.4
0.2
0.0
×103
2
1
0
CX3CR1 expression
0 20 40 60 80 100
 Time, months
PLATINUM TREATED
GEMCITABINE TREATED
1.0
0.8
0.6
0.4
0.2
0.0
×103
8
4
0
CX3CR1 expression
Post-Progression Survival
0 50 100 150 200
 Time, months
1.0
0.8
0.6
0.4
0.2
0.0
×103
8
4
0
CX3CR1 expression
0 50 100 150
 Time, months
| POST-PROGRESSION SURVIVAL | | | POST-PROGRESSION SURVIVAL | | | POST-PROGRESSION SURVIVAL | | |
| --- | --- | --- | --- | --- | --- | --- | --- | --- |
| Serous ov.ca. Platinum treated n=702 | Expression of CX3CR1 | | Serous ov.ca. Gemcitabine treated n=106 | Expression of CX3CR1 | | Serous ov.ca. Topotecan treated n=104 | Expression of CX3CR1 | |
| | LOW | HIGH | | LOW | HIGH | | LOW | HIGH |
| # of subjects | 522 | 180 | # of subjects | 79 | 27 | # of subjects | 66 | 38 |
| PPS, months | 42.13 | 36.80 | PPS, months | 50.53 | 32.50 | PPS, months | 45.53 | 38.47 |
