# Supplementary material for: Regulation of DNA damage repair and lipid uptake by CX3CR1 in epithelial ovarian carcinoma
Source: Oncogenesis. 2018 May 1;7(5):37. doi: 10.1038/s41389-018-0046-6 (PMC5928120; doi:10.1038/s41389-018-0046-6)
Supplement: Supplementary file 12 — supplementary figure 10 [file 41389_2018_46_MOESM12_ESM.pptx]

## Slide 1
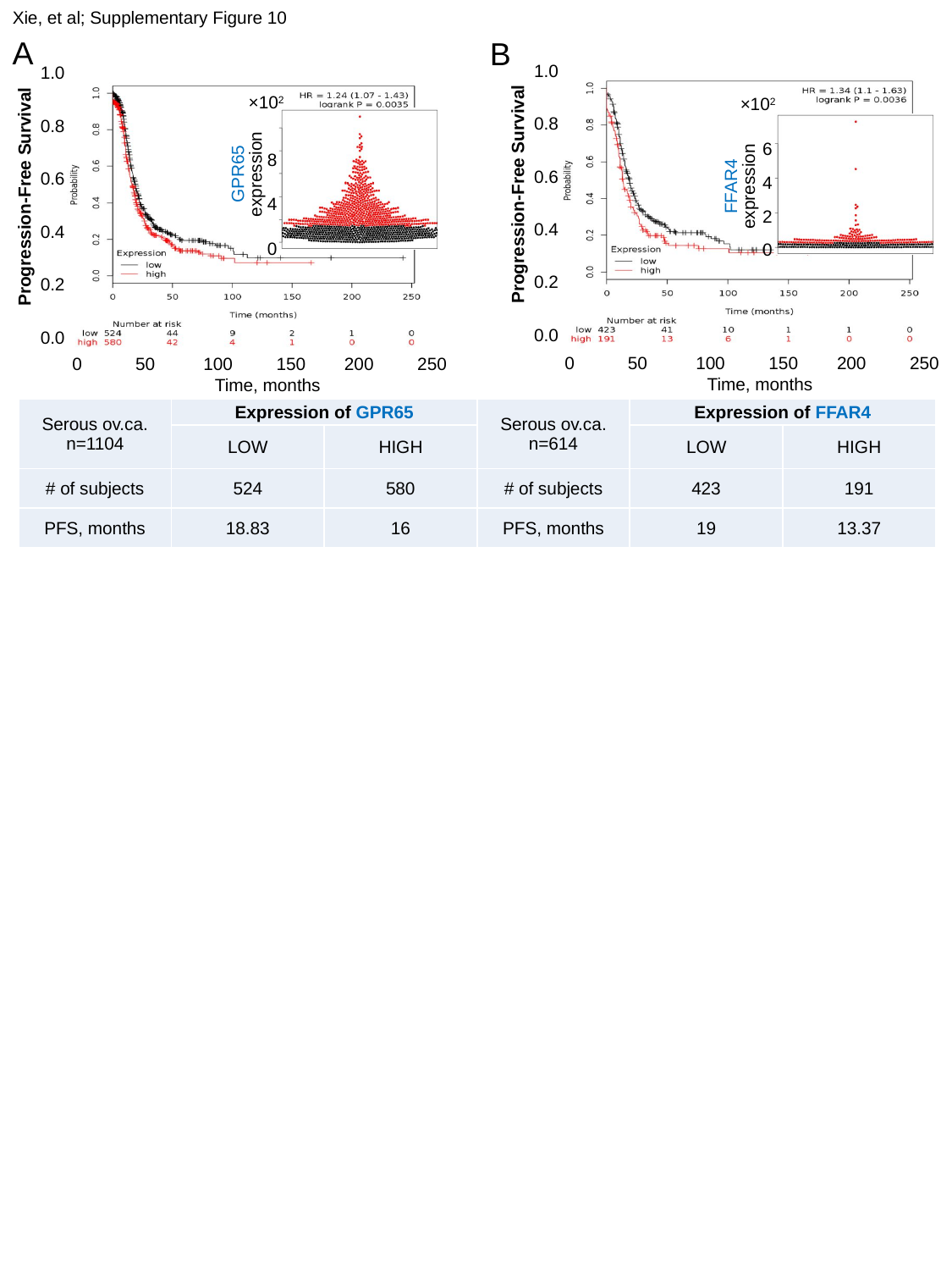

Xie, et al; Supplementary Figure 10
1.0
0.8
0.6
0.4
0.2
0.0
1.0
0.8
0.6
0.4
0.2
0.0
A
B
×102
×102
6
4
2
0
8
4
0
GPR65 expression
FFAR4 expression
Progression-Free Survival
Progression-Free Survival
0 50 100 150 200 250
Time, months
0 50 100 150 200 250
Time, months
| Serous ov.ca. n=1104 | Expression of GPR65 | | Serous ov.ca. n=614 | Expression of FFAR4 | |
| --- | --- | --- | --- | --- | --- |
| | LOW | HIGH | | LOW | HIGH |
| # of subjects | 524 | 580 | # of subjects | 423 | 191 |
| PFS, months | 18.83 | 16 | PFS, months | 19 | 13.37 |
